# Influences of Electromagnetic Energy on Bio-Energy Transport through Protein Molecules in Living Systems and Its Experimental Evidence

**DOI:** 10.3390/ijms17081130

**Published:** 2016-07-25

**Authors:** Xiaofeng Pang, Shude Chen, Xianghui Wang, Lisheng Zhong

**Affiliations:** 1 Institute of Physical Electrons, University of Electronic Science and Technology of China, Chengdu 610054, China; 2 Department of Physics, East China Normal University, Shanghai 200062, China; sdchen@phy.ecnu.edu.cn (S.C.); xhwang@phy.ecnu.edu.cn (X.W.); 3 State Key Laboratory of Electrical Insulation and Power Equipment, Xi’an Jiaotong University, Xi’an 710049, China; lszhong@mail.xjtu.edu.cn

**Keywords:** electromagnetic energy, dipole–dipole interaction, protein molecule, soliton, collagen, bio-energy transport mechanism

## Abstract

The influences of electromagnetic fields (EMFs) on bio-energy transport and its mechanism of changes are investigated through analytic and numerical simulation and experimentation. Bio-energy transport along protein molecules is performed by soliton movement caused by the dipole–dipole electric interactions between neighboring amino acid residues. As such, EMFs can affect the structure of protein molecules and change the properties of the bio-energy transported in living systems. This mechanism of biological effect from EMFs involves the amino acid residues in protein molecules. To study and reveal this mechanism, we simulated numerically the features of the movement of solitons along protein molecules with both a single chain and with three channels by using the Runge–Kutta method and Pang’s soliton model under the action of EMFs with the strengths of 25,500, 51,000, 76,500, and 102,000 V/m in the single-chain protein, as well as 17,000, 25,500, and 34,000 V/m in the three-chain protein, respectively. Results indicate that electric fields (EFs) depress the binding energy of the soliton, decrease its amplitude, and change its wave form. Also, the soliton disperses at 102,000 V/m in a single-chain protein and at 25,500 and 34,000 V/m in three-chain proteins. These findings signify that the influence of EMFs on the bio-energy transport cannot be neglected; however, these variations depend on both the strength and the direction of the EF in the EMF. This direction influences the biological effects of EMF, which decrease with increases in the angle between the direction of the EF and that of the dipole moment of amino acid residues; however, randomness at the macroscopic level remains. Lastly, we experimentally confirm the existence of a soliton and the validity of our conclusion by using the infrared spectra of absorption of the collagens, which is activated by another type of EF. Thus, we can affirm that both the described mechanism and the corresponding theory are correct and that EMFs or EFs can influence the features of energy transport in living systems and thus have certain biological effects.

## 1. Introduction

Prevalent in our environment, electromagnetic fields (EMFs) or waves (EMWs) of varying frequencies and strengths are generated by such sources as the irradiations of high-voltage transmission lines, electrical appliances, microwave stations, and radio equipment. As such, it is necessary to understand whether these externally applied EMFs, EMWs, or electric fields (EFs) have any biological effects on animals and humans [1,2]. Much research has focused on determining any relationship between EMFs and biological processes [1,2,3,4,5,6,7,8,9,10,11,12,13,14,15,16,17,18,19,20,21,22,23]. Li et al. [10,11,12,13,14,15] studied the biologic effects of environmental EMFs, observing the variations of the dielectric constant and conductivity and absorbed strength of infrared light of bio-tissues as well as of the rheology features of the blood in animals under the influences of different electromagnetic fields relative to those without electromagnetic fields. Zhang et al. [16,17] measured the expression of matrix metalloproteinase and tight-junction proteins in rats by following a pulse EMF at high frequencies, which induced blood–brain barrier permeability changes, and also studied the effects of a pulse EMF with high frequencies on the fluorescence spectrum of serum in the rats. Beebe et al. [18] studied the nanosecond pulsed electric field effect on cell growth and the development of bio-tissues, as well as apoptosis induction and tumor growth inhibition. Lazzaro et al. [19] and Apollonio et al. [20] discussed the feasibility of microwave energy affecting biological systems via nonthermal mechanisms and offered systematic approaches, respectively. Hofmann et al. [21] investigated the possibility of electroporation therapy for head and neck cancer. Kekez et al. [22] researched contributions to the biophysics of lethal effects of EFs on microorganisms. Pang et al. [23] investigated and discussed the mechanism and properties of biological thermal effects of microwaves and noted that microwaves can influence the proliferations of cells and tissues. These results indicated that EMFs and EFs have certain biological effects. Although animals or humans are not measurably affected by EMFs unless they come in close contact with high-voltage transmission lines, research should focus more on the proliferation of microwaves and radio waves and their influences on health. Hence, the biological effects of these influences on the health of human beings merit examination.

Only a few conclusions supporting the theory that EMFs and EFs containing microwave radio waves have biological effects have been accepted. Because of varying types of research and instrumentation, experimental results for the same questions range considerably. Thus, a widely accepted and unified conclusion is difficult to acquire, and the mechanism of influence of EMFs on life activity has not been revealed or elucidated [1,2,3]. However, epidemiological investigations have shown that EMFs, including microwaves, radio waves, and those from high-voltage transmission lines, have always had adverse effects on health [3,4,5,6,7,8,9,10,11,12,13,14,15,16]. Therefore, the influence of EMFs on the health of human beings and animals cannot be dismissed. The rapidly increasing usage of electrical appliances, microwave instruments, cell phones, and other electromagnetic devices has led to expansive distribution of EMFs in our environment. In this case, it is necessary to study and release the biological effects of EMFs and their influences on the health of human beings and animals. This need suggests that investigations should focus on the biological effects of EMFs—in particular, the mechanisms in depth through the latest ideas and methods in theoretical analyses and experimental measurements.

One key issue would be to analyze and investigate the electromagnetic properties of biotissues and biomacromolecules and the distributions and properties of charged groups, or atoms and molecules, as well as any variations under the influence of externally applied EMFs. Examination of these charged particles, molecules, or species, as well as their properties of movement, could lead to a better understanding of biological mechanisms under the effect of EMFs. In practice, these charged particles, molecules, or species exist widely in cells and biomacromolecules, such as protein and DNA [9,24], upon which the externally applied EMF can influence their biological processes and properties, and any biological effects can also be exhibited clearly. In this paper, we first seek any mechanism of biological effect from EMFs and EFs. Energy transport, released from the hydrolysis reaction of adenosine triphosphate (ATP) molecules along the protein molecules, is a typical target that exemplifies this described mechanism; i.e., EMFs can disturb and influence energy transport considerably through its interaction with the amino acid residues with certain electric dipole moments. Here we reveal the mechanism of influence of EMFs on energy transport in protein molecules and further study the properties of this mechanism.

## 2. Theories of Energy Transport in Protein Molecules and the Mechanism of Influence of EFs on Transport

### 2.1. Davydov’s Theory of Energy Transport and Its Features

Energy in living systems comes mainly from the hydrolysis reaction of ATP molecules, which is denoted [9,24] by

$${\text{ATP}}^{4-}+{\text{H}}_{2}O\to {\text{ADP}}^{3-}+{\text{HPO}}_{4}^{2-}+H^{+}+0.43eV$$

where ADP is adenosine diphosphate. The energy of 0.43 eV released from this chemical reaction is used in many life activities and processes, such as muscle contraction, neuroelectric pulse transfer along the neurolemma, DNA reduplication, and calcium and sodium pumping in cell membranes, which are some basic life processes and activities in life systems. This function suggests clearly that the energy released in this reaction and its transport is related closely to the growth and development of human beings and animals. Thus, most life processes require the release of energy by ATP hydrolysis, with protein molecules playing a key role in energy transport.

**Figure 1:**
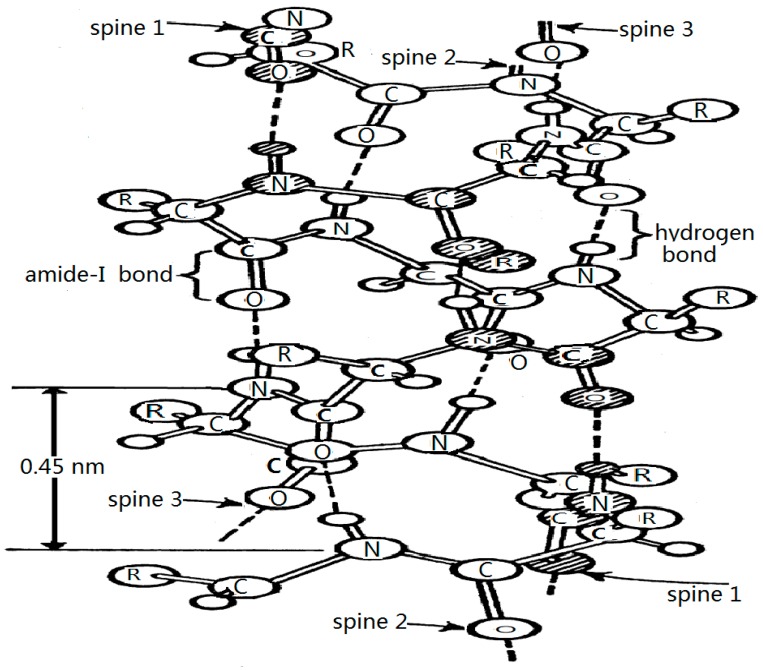
The molecular structure of an α-helical protein molecule.

As it is known, the amino acid molecule consists of an amino group (NH_2_), a carboxyl group (COOH), and a side group or chain functional group attached to an α-carbon atom, they polymerize to form long chains of residues of …H–N–C=O…H–N–C=O…H–N–C=O…H–N–C=O…, where the dotted lines indicate the hydrogen bond, that constitute the chain structure of protein molecules. When the polypeptide chains have been formed, they can further fold into an α-helix, a β-sheet, and a globular conformation. In an α-helix structure, three chains of hydrogen-bonded peptide groups can be represented approximately along the horizontal direction of the sequence. In this process, the distributions of the positive and negative charges in each amino acid residue change, and most of the amino acid residues are polarized and have certain electric dipole moments. Figure 1 shows a typical structure of an α-helix protein molecule [24,25,26,27,28,29,30,31,32,33], in which the forms of the three polypeptide chains built by hydrogen bonds and amide-I bonds are clearly exhibited.

However, questions remain as to how bio-energy is transported into life systems. Although several decades of research have been amassed, in 1971 Davydov proposed the soliton theory. According to this theory, bio-energy transports along the α-helical protein molecules, for which the structure is denoted in Figure 1 [24,25,26,27,28,29,30,31,32,33]. The mechanism of the bio-energy transport can be described as follows. The stretching vibration of C=O bonds(Amide Is) in the amino acid residues in the proteins is affected by energy released from ATP hydrolysis, in which the vibrational quanta, called excitons, and the deformation of amino acid residues also occur simultaneously and influence each other in this case. Thus, the nonlinear coupling interaction between the excitons and the deformation of amino acid residues, that occur in this process, make the excitons self-trap as a soliton, which transports further along the protein molecules in virtue of the dipole–dipole interaction between neighboring amino acid residues. This is a well-known soliton mechanism of energy transport in life systems. Davydov’s theory works in α-helix protein molecules with three channels in Figure 1 [24,25,26,27,28,29,30,31,32,33]. In this case, a biological process of energy transport always occurs from the produced place to the absorbed place in the living system, which is carried out by means of protein molecules such as collagen, myosin, myoglobin, and actin.

According to the mechanism of bio-energy transport, Davydov established the theory of bio-energy transport in protein molecules [24,25,26,27,28,29,30,31,32,33], in which Davydov gave the Hamiltonian of the protein molecules, which was represented by

(1) $$\begin{array}{l} H_{D}=\sum_{n}[\varepsilon_{0}B_{n}^{+}B_{n}-J(B_{n}^{+}B_{n}+B_{n}B_{n}^{+})]+\sum_{n}[\frac{P_{n}^{2}}{2M}+\frac{1}{2}w{\left(u_{n}-u_{n-1}\right)}^{2}]\\ +\sum_{n}[\chi_{1}(u_{n+1}-u_{n-1})B_{n}^{+}B_{n}]=H_{ex}+H_{ph}+H_{\mathrm{int}} \end{array}$$

where $B_{n}^{+}(B_{n})$ is the creation (annihilation) operator for an Amide I quantum (exciton) in the site *n*, *u_n_* is the displacement operator of amino acid residue at site *n*, *P_n_* is its conjugate momentum operator, *M* is the mass of an amino acid residue and *M* = 1.17 × 10^−25^ kg in single-protein molecules or 5.73 × 10^−25^ kg in α-helix proteins with three channels, w is the elastic constant of the protein molecular chains and w = (13–19.5) N/m for single-protein molecules or (39–58.5) N/m for α-helix proteins, $\chi_{1}$ = 6.2 × 10^−11^ N is a nonlinear coupling parameter and represents the size of the exciton-phonon interaction in this process, $\varepsilon_{0}$ = 0.205 eV is the energy of the Amide I quantum (exciton), J is the dipole–dipole interaction energy between neighboring amino acid residues, J = 1.55 × 10^−22^ or J = 9.68 × 10^−4^ eV, and $r_{0}$ = 4.5 × 10^−10^ m is the average distance between the neighboring amino acid residues [24,25,26,27,28,29,30,31,32,33].

The wave function of the systems proposed by Davydov [24,25,26,27,28,29] is the form of

(2) $$D_{2}(t)=|\varphi_{D}\rangle |\beta (t)\rangle =\sum_{n}\varphi_{n}(t)B_{n}^{+}\exp\{-\frac{i}{\hbar }\sum_{n}[\beta_{n}(t)P_{n}-\pi_{n}(t)u_{n}]\}|0\rangle$$

where $|0\rangle ={|0\rangle }_{ex}{|0\rangle }_{ph}$, ${|0\rangle }_{ex}$ and ${|0\rangle }_{ph}$ are the ground states of the exciton and phonon, respectively, Davydov’s soliton, obtained from Equations (1) and (2) in the semiclassical limit and using the continuum approximation [24,25,26,27,28,29], has the form of

(3) $$\varphi_{D}(x,t)={\left(\frac{u_{D}}{2}\right)}^{1/2}\text{ seach}\left[\frac{\mu_{D}}{r_{0}}(x-x_{0}-vt)\right]\exp\left\{i\left[\frac{\hbar v}{2Jr_{0}^{2}}(x-x_{0})-E_{v}t/\hbar \right]\right\}$$

If, $|\varphi_{D}(t)\rangle =\sum_{n}\varphi_{n}(t)B_{n}^{+}{|0\rangle }_{ex}$ in Equation (2) is an eigenstate of the number operator, $\overset{\wedge }{N}=\sum_{n}B_{n}^{+}B_{n}$, then the soliton contains only one exciton because $N=\langle \varphi_{D}(t)|\overset{\wedge }{N}|\varphi_{D}(t)\rangle =1$, i.e., $\overset{\wedge }{N}|\varphi_{D}(t)\rangle =1|\varphi_{D}(t)\rangle$. This finding indicates that the Davydov soliton contains only one excitation, which corresponds to the excited state of a single particle and is localized over a scale of $r_{0}/\mu_{D}$; where $\mu_{D}=\chi_{2}^{2}/(1-s^{2})wJ,s^{2}=v^{2}/v_{0}^{2}$, and $v_{0}=r_{0}{\left(w/M\right)}^{1/2}$; where $v_{0}$ is the sound speed in the protein molecular chains; *v* is the velocity of the soliton; and $G_{D}=4J\mu_{D}$ is its nonlinear interaction energy accepted in this process. This finding shows that Davydov’s soliton is formed through the self-trapping of one exciton and that it has a binding energy $E_{BD}=-\chi_{1}^{4}/3Jw^{2}$.

These results clearly exhibit that the energy released from the hydrolysis reaction of the ATP molecule promoted the form of a soliton and its transported along the protein molecules in a bell-type solitary wave or a soliton with an invariable amplitude and velocity, as given in Equation (3) [9,27,34]. This finding indicates that the energy cannot be damped and dissipated in the transport process and is significant for biological processes because energy is retained in the transport process due to the feature and essence of the soliton. Thus, these life activities clearly require the soliton.

Davydov’s idea yields a compelling picture of the mechanism of energy transport in living systems and has led to extensive research in biophysics [34,35,36,37,38,39,40,41,42,43,44,45,46,47,48,49,50,51,52,53,54,55,56,57,58,59,60,61,62,63,64,65,66,67,68,69,70,71,72,73]. However, the issues related to Davydov’s model, including its foundation, accuracy quantum and classical properties, thermal stability, and lifetimes, have been the focus of much research [34,35,36,37,38,39,40,41,42,43,44,45,46,47,48,49,50,51,52,53,54,55,56,57,58,59,60,61,62,63,64,65,66,67,68,69,70,71]. These investigations and discussions focus mainly on the validity of the Davydov theory and the thermal stability of the Davydov soliton at the biological temperature 300 K. Some numerical simulations have indicated that the Davydov soliton is not stable at this temperature [36,37,38,39,40,41,42,43,44,45,46,47]. At the same time, Monte Carlo numerical calculations indicate that the correlation characteristic of soliton-like quasiparticles occurs only at low temperatures [42,43,44], approximately *T* < 10 K, for widely accepted parameter values. This finding is consistent at a qualitative level with the results of Cottingham et al. [46] and Schweitzer [47]. The latter is a straightforward quantum-mechanical perturbation calculation, in which the lifetime of the Davydov’s soliton is approximately 10^−12^–10^−13^ s at 300 K, in which the soliton can transport only over approximately 10 amino acid residues. Therefore, Davydov’s theory is not suitable for protein molecules. In addition, Förner’s investigations showed that Davydov’s soliton is stable only at 40 K and that it disperses completely at higher temperatures [39,40,41,42,43]. These results indicate clearly that Davydov’s soliton is not a real carrier of the energy transport in protein molecules; thus, Davydov’s theory is not appropriate to the systems. This finding demonstrates the necessity of developing new theories of energy transport in living systems.

### 2.2. Pang’s Theory of Energy Transport and Its Properties

On the basis of the difficulties described concerning Davydov’s theory and the results researched by Cruzeiro-Hansson [37,38] and Förner et al. [39,40,41,42,43,44,45,46,47,48,49,50,51,52,53,54,55,56,57,58,59,60,61,62,63,64,65,66,67,68,69,70,71,72,73,74], we improve Davydov’s model by changing simultaneously the Hamiltonian and the wave function of the systems, in which we added a new coupling interaction of the excitons with the displacement of amino acid residues into the Hamiltonian in Equation (1) and replaced further the Davydov’s wave function of the one-quantum (exciton)excited state in Equation (2) by a quasi-coherent two-quantum state [75,76,77,78,79,80,81,82,83,84,85,86,87,88,89,90,91,92,93,94,95,96,97,98,99,100,101,102]. In this case, the representations in Equations (1) and (2) for the single-channel protein molecules are replaced by

(4) $$\begin{array}{l} |\Phi (t)\rangle =|\alpha (t)\rangle |\beta (t)\rangle =\frac{1}{\lambda }[1+\sum_{n}\alpha_{n}(t)B_{n}^{+}+\frac{1}{2!}{\left(\sum_{n}\alpha_{n}(t)B_{b}^{2}\right)}^{2}]{|0\rangle }_{ex}\times \\ \exp\{-\frac{i}{\hbar }\sum_{n}[\beta_{n}(t)P_{n}-\pi_{n}u_{n}{|0\rangle }_{ph} \end{array}$$

and

(5) $$\begin{array}{l} H=H_{ex}+H_{ph}+H_{\mathrm{int}}=\sum_{n}[\varepsilon_{0}B_{n}^{+}B_{n}-J(B_{n}^{+}B_{n}+B_{n}B_{n}^{+})]+\sum_{n}[\frac{P_{n}^{2}}{2M}+\frac{1}{2}w{\left(u_{n}-u_{n-1}\right)}^{2}]+\\ \sum_{n}[\chi_{1}(u_{n+1}-u_{n-1})B_{n}^{+}B_{n}+\chi_{2}(u_{n+1}-u_{n})(B_{n+1}^{+}B_{n}+B_{n}^{+}B_{n+1})] \end{array}$$

respectively; where the created and annihilated operators of the exciton are represented by $B_{n}^{+}$ and $B_{n}$, respectively; ${|0\rangle }_{ex}$ and ${|0\rangle }_{ph}$ are the ground states of the exciton and phonon, respectively; and *u*_n_ and *P*_n_ are the displacement and momentum operators of amino acid residue at the site *n*, respectively. The $\beta_{n}(t)=\langle \Phi (t)|u_{n}|\Phi (t)\rangle$, and $\pi_{n}(t)=\langle \Phi (t)|P_{n}|\Phi (t)\rangle$ are two sets of unknown functions; and *λ* is a normalization constant. Present nonlinear coupling constants are $\chi_{1}$ and $\chi_{2}$ = (10−15) × 10^−11^ N, which represent the modulations of the on-site energy and dipole–dipole interaction energy of excitons due to the variations of displacements of amino acid residue in the protein molecules, respectively. Other parameters are same as those in the Davydov‘s model mentioned previously.

Using Equations (4) and (5) and working from the Schrödinger equation and the Heisenberg equation, we obtained

(6) $$\begin{array}{l} i\hbar {\dot{\alpha }}_{n}(t)=\varepsilon_{0}\alpha_{n}(t)-J[\alpha_{n+1}(t)+\alpha_{n-1}(t)]+\chi_{1}[q_{n+1}(t)-q_{n-1}(t)]\alpha_{n}(t)+\\ \chi_{2}[q_{n+1}(t)-q_{n}(t)][\alpha_{n+1}(t)+\alpha_{n-1}(t)]+\\ \frac{5}{2}\{w(t)-\frac{1}{2}\sum_{m}q_{m}(t)\pi_{m}(t)-{\dot{\pi }}_{m}(t){\dot{q}}_{m}(t)]\}\alpha_{n}(t) \end{array}$$

(7) $$\begin{array}{l} M{\overset{..}{q}}_{n}(t)=W[q_{n+1}(t)-2q_{n}(t)+q_{n-1}(t)]+2\chi_{1}[{\left|\alpha_{n+1}\right|}^{2}-\left|\alpha_{n-1}(t)\right|]+\\ 2\chi_{2}\{\alpha_{n}^{*}(t)[\alpha_{n+1}(t)-\alpha_{n-1}(t)]+\alpha_{n}(t)[\alpha_{n+1}^{*}(t)-\alpha_{n-1}^{*}(t)]\} \end{array}$$

In the continuum approximation, we get from Equations (6) and (7) [73,74,75,76,77,78,79,80,81,82,83,84]

(8) $$i\hbar \frac{\partial }{\partial t}\alpha (x,t)=R(t)\alpha (x,t)-Jr_{0}^{2}\frac{\partial^{2}}{\partial x^{2}}\alpha (x,t)-G_{p}{\left|\alpha (x,t)\right|}^{2}\alpha (x,t)$$

and

(9) $$M\frac{\partial^{2}\beta (x,t)}{\partial t^{2}}-wr_{o}^{2}\frac{\partial^{2}\beta (x,t)}{\partial x^{2}}=-4\left(\chi_{1}+\chi_{1}\right)r_{0}\frac{\partial }{\partial x}{\left|\alpha (x,t)\right|}^{2}$$

where $R(t)==\varepsilon_{0}-2J+\frac{5}{2}\{W(t)-\frac{1}{2}\sum_{m}\left[{\dot{\beta }}_{n}(t)\pi_{m}(t\right)-{\dot{\pi }}_{n}(t)\beta (t)]\}$.

The soliton solution of Equation (8) is denoted by

(10) $$\alpha (x,t)={\left(\frac{\mu_{p}}{2}\right)}^{1/2}\sec h\left[\left(\mu_{p}/r_{0}\right)\left(x-x_{0}-vt\right)\right]\times \exp\left\{i\left[\frac{\hbar v}{2Jr_{0}^{2}}\left(x-x_{0}\right)-E_{v}\frac{t}{\hbar }\right]\right\}$$

With $\mu_{P}=\frac{2{\left(\chi_{1}+\chi_{2}\right)}^{2}}{w(1-s^{2})J}$ and the nonlinear interaction en $G_{P}=\frac{8{\left(\chi_{1}+\chi_{2}\right)}^{2}}{w(1-s^{2})}$ and $s=v/v_{0}$.

In this case, the energy of the soliton in Equation (10), or the energy transported by the soliton, is obtained by

(11) $$\begin{array}{ll} E=< & \Phi (t)|H|\Phi (t)>=\frac{1}{r_{0}}\int_{-\infty }^{\infty }2[Jr_{0}^{2}{\left(\frac{\partial \alpha }{\partial x}\right)}^{2}+R{\left|\alpha (x,t)\right|}^{2}-G_{p}{\left|\alpha (x,t)\right|}^{4}dx+\\ & \frac{1}{r_{0}}\int_{-\infty }^{\infty }\frac{1}{2}[M{\left(\frac{\partial \beta (x,t)}{\partial t}\right)}^{2}+wr_{0}{\left(\frac{\partial \beta (x,t)}{\partial x}\right)}^{2}]dx=E_{0}+\frac{1}{2}M_{sol}v^{2}. \end{array}$$

The static (rest) energy of the soliton is

(12) $$E_{0}=2(\varepsilon_{0}-2J)-\frac{8{\left(\chi_{1}+\chi_{2}\right)}^{4}}{3w^{3}J}=E_{s}^{0}+W$$

where $W=[2{\left(\chi_{1}+\chi_{2}\right)}^{4}]/3w^{2}J$ is the energy of deformation of the amino acid residues. The effective mass of the soliton is

(13) $$M_{sol}=2m_{ex}+\frac{8{\left(\chi_{1}+\chi_{2}\right)}^{4}(9s^{2}+2-3s^{4})}{3w^{2}J{\left(1-s^{2}\right)}^{3}v_{0}^{2}}$$

In such a case, the binding or forming energy of the soliton in Pang’s theory is denoted by

(14) $$E_{BP}=\frac{-8{\left(\chi_{1}+\chi_{2}\right)}^{4}}{3Jw^{2}}$$

These mathematical models clearly indicate that the properties of Pang’s soliton [75,76,77,78,79,80,81,82,83,84,85,86,87,88,89,90,91,92,93,94,95,96,97,98,99,100,101] differ from those of Davydov’s. Through concrete calculations, we found that Pang’s soliton is thermally stable and has a sufficient lifetime of 10^−9^–10^−10^ s at the physiological temperature of 300 K, which is approximately 800× that of Davydov’s soliton (10^−12^−10^−13^ s), in which Pang’s soliton can transport more than several hundreds of amino acid residues. Thus, Pang’s soliton plays an important role in the biological processes. The comparison of Pang’s soliton with Davydov’s soliton is given in Table 1, which clearly indicates that Pang’s soliton (or theory) differs considerably from that of Davydov’s soliton (or theory). Since then, the results of Pang’s theory were also confirmed by other studies [75,76,77,78,79,80,81,82,83,84,85,86]. Therefore, Pang’s soliton is a carrier of energy transport, and the Pang’s model can be applied to energy transport in protein molecules [97,101,102,103,104,105,106]. Thus, we here applied the Pang’s model to study the influences of EFs and EMFs on properties of energy transport in α-helical protein molecules (Figure 1).

### 2.3. Mechanism of Influence of Electric Field on Energy Transport in Protein Molecules

Equations (11)–(14) indicate that the properties of the energy transported by the soliton involving its velocity (v), effective mass ($M_{sol}$), energy (*E*), and rest energy (*E*_0_), which are determined by the features of the exciton, the amino acid residues, and the structure of the protein molecule, including the effective mass of the exciton (*m*_ex_), the mass of the amino acid residue (*M*), the elastic constant of the protein molecular chains (*w*), the nonlinear exciton–phonon interaction coefficients ($\chi_{1}$ and $\chi_{2}$), the Amide I vibrational energy ($\varepsilon_{0}$), the dipole–dipole interaction energy between the neighboring amino acid residues (*J*), and the distance between the neighboring amino acid residues ($r_{0}$). This clearly indicates that the properties of the energy transport or the soliton will change once these characteristic parameters of the exciton, amino acid residues, and protein molecule structures are altered under the influences of externally applied electromagnetic or light fields, as well as temperatures. On the basis of these characters and features, we seek the mechanism that influences the EMFs or the EFs on energy transport in protein molecules.

As described, energy released from the hydrolysis reaction of the ATP molecules is transported along protein molecules in the solitons by virtue of the dipole–dipole interaction between neighboring amino acid residues with certain electric dipole moments. The externally applied EFs or EMFs can interact with these amino acid residues according to the EMF theory; thus, the properties of the energy transport or movement of soliton will vary, along with the externally applied EFs or EMFs, because the latter can vary the sizes and directions of the dipole–dipole interaction between the neighboring amino acid residues in such a case. Thus, some biological effects arising from the variations of the biological energy in protein molecule will occur correspondingly, which is the influencing mechanism of EMF or EF on the energy transport. In other words, this is a mechanism of biological effect through EMFs or EFs.

If we assume that the strength of the EF in the EMF is denoted by $\vec{E}$ and that the electric dipole moments of the amino acid residue in the protein molecules is denoted by $\vec{p}$, then the energy of interaction of the EF or the EMF with the amino acid residues can be denoted by $\vec{E}.\vec{p}$, according to the EMF theory [82,87,99]. In such a case, the dipole–dipole interaction energy between neighboring amino acid residues in the protein molecules will be changed from *J* to $J+\vec{E}.\vec{p}$. Then we can affirm that the properties of the energy transported by the soliton will be altered notably because the soliton in Equations (10)–(14) is very sensitive to variations of the dipole–dipole interaction between neighboring amino acid residues. This conclusion will be confirmed by the following results.

## 3. Variation of Properties of Energy Transport Arising from the EF in Protein Molecules

### 3.1. Analytic Results for Changes of Properties of the Soliton Transporting the Energy under the Influence of EFs

As mentioned previously, when protein molecules are exposed in an EMF or an EF, the dipole–dipole interaction between the neighboring amino acid residues in the protein molecules is changed into $J+\vec{E}.\vec{p}$. Then the variation of features of the solitons in Equations (10)–(14) in Pang’s model will appear in this case [75,76,77,78,79,80,81,82,83,84,85,86]. These variations in the soliton from Equations (10)–(14) can be obtained, which are described as follows:

(1) The amplitude ($\mu_{P}$) effective mass (*M*_sol_), energy (*E*), rest energy (*E*_0_), and binding energy *E_BP_* of the soliton are decreased and depressed after EF is applied because the physical parameters in Equations (11)–(14) are inversely proportional to the dipole–dipole interactional energy *J*. This means that the capability and value of the energy transported by the soliton is decreased because of the increases of dipole–dipole interaction between neighboring amino acid residues. If the strength of an EF in an EMF is very high, then the capability and value of the energy transport will depress considerably. In such a case, we could infer and suppose that some new biological effects will occur and that these new biological effects have just arisen from the EF
(2) The EF varies the form and outline of the soliton in Equation (10) because the form and outline of the solitary wave, $Sech[(\mu_{P}/r_{0})(x-x_{0}-vt)]\times \exp\{i[\frac{\hbar v}{2Jr_{0}^{2}}(\chi_{1}+\chi_{2})-E_{v}\frac{t}{\hbar }]\}$, in Equation (10) and its amplitude of envelope, $\mu_{P}/r_{0}$, as well as its phase of the carrier wave, $\left[\frac{\hbar v}{2Jr_{0}^{2}}(x-x_{0})-E_{v}\frac{t}{\hbar }\right]$, are all changed with the variation of dipole–dipole interaction *J* under the influenceof EF. These changes arising from EFs will also affect the proliferation of cells and life bodies because of variations in the bio-energy they obtain.
(3) The biological effects of EFs closely depend on both strength and direction with respect to the dipole moment of amino acid residues because the externally applied electric-field, $\vec{E}$ and dipole moments of the amino acid residue, $\vec{p}$, all possess a certain strength and direction. In this case, we should consider the direction and strength of the EF, $\vec{E}$, and the dipole moments of the amino acid residue as well as their relationships. Thus, the electromagnetic energy of interaction between them should be represented by $\vec{E}.\vec{p}=\left|\vec{E}\right|\left|\vec{p}\right|\cos\theta$, where *θ* is the angle between the two vectors, $\vec{E}$ and $\vec{p}$. This implies that the variations of the dipole–dipole interaction between neighboring amino acid residues caused by the EF should be expressed by $(J+\vec{E}.\vec{p})-J=\left|\vec{E}\right|\left|\vec{p}\right|\cos\theta$, which decreases when the angle (*θ*) increases. If *θ* = 0, then $\vec{E}.\vec{p}=\left|\vec{E}\right|\left|\vec{p}\right|$. If $\theta =90^{0}$, then $\vec{E}.\vec{p}=0$. Therefore, the EF has a stronger biological effect on the former and no biological effect on the latter. This result indicates clearly that the biological effect depends on the direction of externally applied EF with respect to that of electric dipole moments of the amino acid residues. If the directions of EF are different, although their strengths are the same, then their biological effects are also different. This finding implies that the biological effects of EFs depend on both the strength and the direction with respect of the dipole moment of amino acid residues [81,86,97].

However, it is worth noting the direction from which the biological effects arise from the EF at the macroscopic level. The orientations of many proteins and amino acid residues within the proteins over any macroscopic region tend to be random. However, this condition depends on the biotissue structure because some biotissues are well organized. Clearly, the orientation of the EF to the structure will be important in this case. In other cases, the overall effect being calculated will not depend on the direction of the field.

### 3.2. Results of Numerical Simulation for Changes of Property of the Energy Transport Resulting from EF

#### 3.2.1. Results in Single-Protein Chains

We now investigate the influences of EFs in EMFs on the energy transported by the soliton in protein molecules with single channels and three channels by using the numerical simulation method [107,108], respectively. In this case, the variation of the dipole–dipole interaction is represented by $(J+\vec{E}.\vec{p})-J=\left|\vec{E}\right|\left|\vec{p}\right|\cos\theta$, which is related to the strengths of the EF in the EMF. Therefore, our purpose is to determinate the changed features of the movement of the soliton transporting the energy with varying EFs, which can be obtained by the fourth-order Runge–Kutta method [107,108]. In such a case, we must establish the dynamic equations of the soliton in this numerical simulation. Obviously, the dynamic equations of the soliton can be obtained from Equations (6) and (7) in Pang’s model. Their derivations are depicted as follows.

First, we use first the transformation $a_{n}(t)\to a_{n}(t)\exp[i\varepsilon_{0}t/\hbar ]$ to eliminate the term $\varepsilon_{0}a_{n}(t)$ in Equation (6). Then, we make the transformation according to the Runge–Kutta method [107,108]: $a_{n}(t)=a_{n}(t)r_{n}+ia(t)i_{n}$. Thus, Equations (6) and (7) become

(15) $$\hbar \dot{\alpha }r_{n}=-J(\alpha i_{n+1}+\alpha i_{n-1})+\chi_{1}(q_{n+1}-q_{n-1})\alpha i_{n}+\chi_{2}(q_{n+1}-q_{n})(ai_{n+1}+\alpha i_{n-1})$$

(16) $$-\hbar \dot{\alpha }i_{n}=-J(\alpha r_{n+1}+\alpha r_{n-1})+\chi_{1}(q_{n+1}-q_{n-1})\alpha ir_{n}+\chi_{2}(q_{n+1}-q_{n})(ar_{n+1}+\alpha r_{n-1})$$

(17) $${\dot{q}}_{n}=y/M$$

(18) $$\begin{array}{l} \dot{y}=W(q_{n+1}-2q_{n}+q_{n-1})+2\chi_{1}(\alpha r_{n+1}^{2}+\alpha i_{n+1}^{2}-\alpha r_{n-1}^{2}-\alpha i_{n-1}^{2})+\\ 4\chi_{2}[\alpha r_{n}(\alpha r_{n+1}-\alpha r_{n-1})+\alpha i_{n}(\alpha i_{n+1}-\alpha i_{n-1})] \end{array}$$

(19) $${\left|\alpha_{n}\right|}^{2}={\left|\alpha r_{n}\right|}^{2}+{\left|\alpha i_{n}\right|}^{2}$$

where *ar_n_* and *ai_n_* are real and imaginary parts of *a*_n_, respectively.

Equations (15)–(19) are discretely coupled and can be used to determine the dynamic features and states of the soliton by using the fourth-order Runge–Kutta method in the numerical simulation. Obviously, in this case there are four equations for one amino acid residue from Equations (15)–(18). Thus, we should find solutions of 4 N associated equations for the protein molecules constructed by N amino acid residues. However, when the fourth-order Runge–Kutta method [107,108] is used to find the solutions of Equations (15)–(18), we should discretize these equations further. Thus, the *n* is replaced by *j*, the time is denoted by *n*, and the step length of the space variable is denoted by *h* in the previous equations in this simulation. Then we can get the representations of the solutions from the given equations. The concrete equations for finding the solutions are described in Appendix A.

In concrete calculations, we must determine the values of the parameters in Equations (15)–(18). The values for the parameters *M*, *J*, *W*, $\varepsilon_{0}$, *x*_1_, and *x*_2_ are known in Pang’s theory of the energy transport in protein molecules. The widely accepted values of these parameters are J = 9.68 × 10^−4^ eV, $\varepsilon_{0}$ = 0.205 eV, $\chi_{1}=6.2\times 10^{-11}N,w=13\text{ N}/\text{m}$, $\chi_{2}=(10-18)\times 10^{-12}\text{ N}$ and *M* = 1.17 × 10^−25^ kg in single-protein molecules or 5.73 × 10^−25^ kg in α-helix proteins [24,25,26,27,28,29,30,31,32,33,64,65,66,67,68,69,70,71,72,73,74,75,76,77,78,79,80,81,82,83,84,85,86,87,88,89,90]. However, we need to confirm the value of the electric dipole moment $\left|\vec{p}\right|$ of the amino acid residues, which is a key parameter for finding the relationship to varying solitons transporting the bio-energy with changing EFs applied externally to protein molecules. Many researchers have calculated and measured the values of $\left|\vec{p}\right|$ of the amino acid residues in protein molecules [24,109,110,111]. Results have shown the electric dipole moments in the region of a few −10 debyes (1 debye = 3.33 × 10^−30^ cm). Therefore, it is reasonable to choose $\left|\vec{p}\right|=10$ debyes in the calculation [24,109,110,111].

From Equations (15)–(19) using the given values of parameters, we can determine the changes of the solution of Equations (15)–(19) numerically, $a(t)r_{n}$ and $a(t)i_{n}$, and of the corresponding ${\left|a_{n}(t)\right|}^{2}$, which denotes the probability of appearance of the soliton related to its amplitude, with varying time and space by the fourth-order Runge–Kutta method [107,108]. In this calculation, we choose the time step Δt to be between 0.2 and $0.5{\left(\bar{M}/\bar{w}\right)}^{1/2}$ and the initial excitation of $a_{n}(0)=A\sec h[(n-n_{0})\times ({\bar{\chi }}_{1}+{\bar{\chi }}_{2})/4\bar{J}\bar{w}]$ (where *A* is normalization constant) and the initial states of amino acid residues, *q*_n_(0) = π_n_(0) = 0 at the site n; where the molecular chain is fixed, the number of amino acid residues, N, is chosen to be 400 and a time step of 0.0195 is used. This means that 800 coupled differential equations with first-order-of-time derivation had to be solved in such a case. At the same time, the system of units eV for energy, $\overset{0}{A}$ for length, and ps for time have been used to be suitable for the numerical solutions of Equations (15)–(19). Otherwise, by using numerical simulations obtained by the fourth-order Runge–Kutta method [107,108], we could also satisfy the total energy of the soliton, *E* ≤ *Φ*(*t*)|*H*|*Φ*(*t*) ≥ constant, in which a possible imaginary part of the energy, which could occur because of numerical inaccuracies, is zero. Its accuracy is 0.001 feV; when the soliton is in motion, its probability of appearance must be normalized at all times, i.e., the number of particles for the system must be conservative, ${\sum_{n}\left|a_{n}(t)\right|}^{2}={\sum_{n}\left|a_{n}(0)\right|}^{2}=1$ up to 0.3 ppm. Meanwhile, the initial excitation conditions described previously are also required in this calculation. Total numerical simulation is performed through data-paralleling algorithms and *MATLAB* language. On the basis of the methods and conditions given, we obtain the representation for the change of |*a_n_*|^2^=|*a_j_*|^2^, which is the probability of the soliton occurred at the *n*th (or *j*th) amino acid molecule, with varying positions and times under the actions of different EFs.

**Figure 2:**
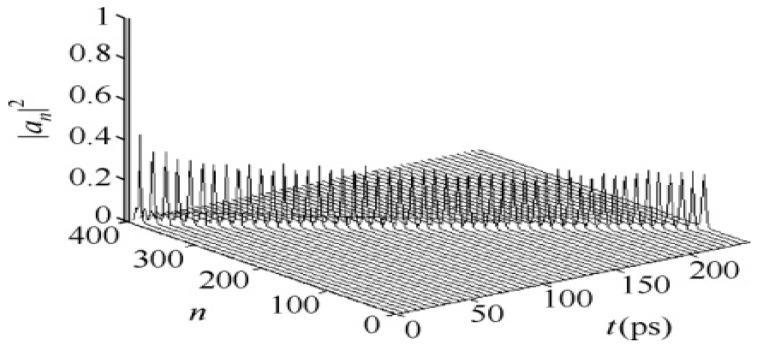
State of the new soliton in the case of a longtime period of 250 ps and long spacing of 400 amino acid residues.

**Figure 3:**
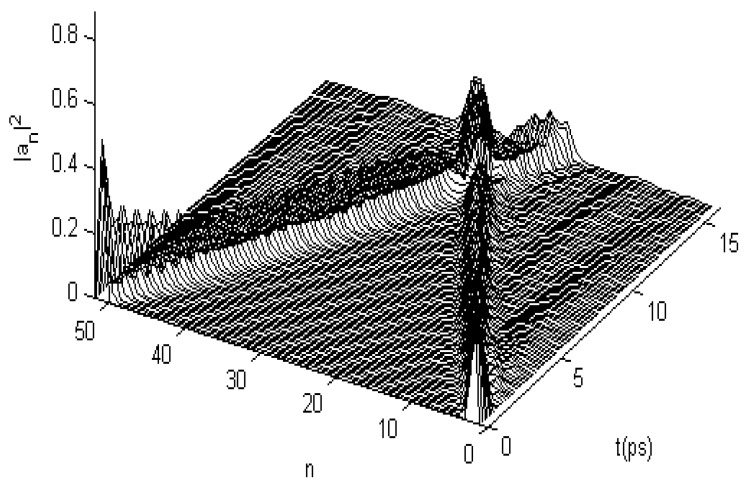
The collision behavior of two solitons for Equations (18)–(21).

We first calculate the solutions of Equations (15)–(19) numerically in the uniform and periodic proteins with a single chain using the above-average values for these parameters, i.e., $\bar{M}$ = 1.17 × 10^−25^ kg; $\bar{J}$ = 1.55 × 10^−22^ J; or = 9.68 × 10^−4^ eV; $\bar{\varepsilon_{0}}$ = 0.20 5 eV; $\bar{\chi_{1}}$ = 6.2 × 10^−11^ N; $\bar{w}$ = (13 − 19.5) N/m; and $\bar{\chi_{2}}=(11-18)\times 10^{-12}\text{ N}$, which are widely accepted in investigations of energy transport in α-helical protein molecules [24,25,26,27,28,29,30,31,32,33,65,66,67,68,69,70,71,72,73,74]. If the fourth-order Runge–Kutta method is used, then the results for the state of transport of the soliton in the time–place are shown in Figure 2 and Figure 3, respectively, in which we show the movements or progress of the soliton transporting the energy (in other words, its probability ${\left|a_{n})t)\right|}^{2}$) with varying time and space, where the symbols of three axes and their physical significances are marked clearly; where *n* is the number of amino acid residues the soliton has transported; and *t* is time of movement of the soliton. If the probabilities or amplitudes of the soliton, obtained from this calculation, are always constant in the movement process, then we can affirm that the solution of the dynamic equations is a soliton. Otherwise, these equations do not provide the soliton solution, which indicates that the soliton was dispersed or damped and that the energy transport was also dispersed or dissipated due to the influence of an externally applied field, according to the soliton theory [9,27,36]. Therefore, we can study and confirm the influences of EFs or EMFs on the soliton or energy transport in the protein molecules through the results obtained from the numerical simulations. Figure 2 and Figure 3 indicate that this solution is a soliton because its probabilities or amplitudes are invariable in the propagation processes of the long-time period of 250 ps and the long distances of 400 amino acid residues, which are shown in Figure 2. Figure 3 exhibits the collision property of two solitons and also shows that the probabilities or amplitudes of the two solitons are invariable after the collision, resembling a feature of classical particles. Therefore, Figure 2 and Figure 3 clearly show that the numerical solutions of Equations (15)–(19) are reliable and indicate the presence of a soliton according to the soliton theory [9,27,34].Thus, we can affirm that Equations (15)–(19) have the soliton solution, which is exactly the carrier of the energy transport in uniform and periodic protein molecules.

**Figure 4:**
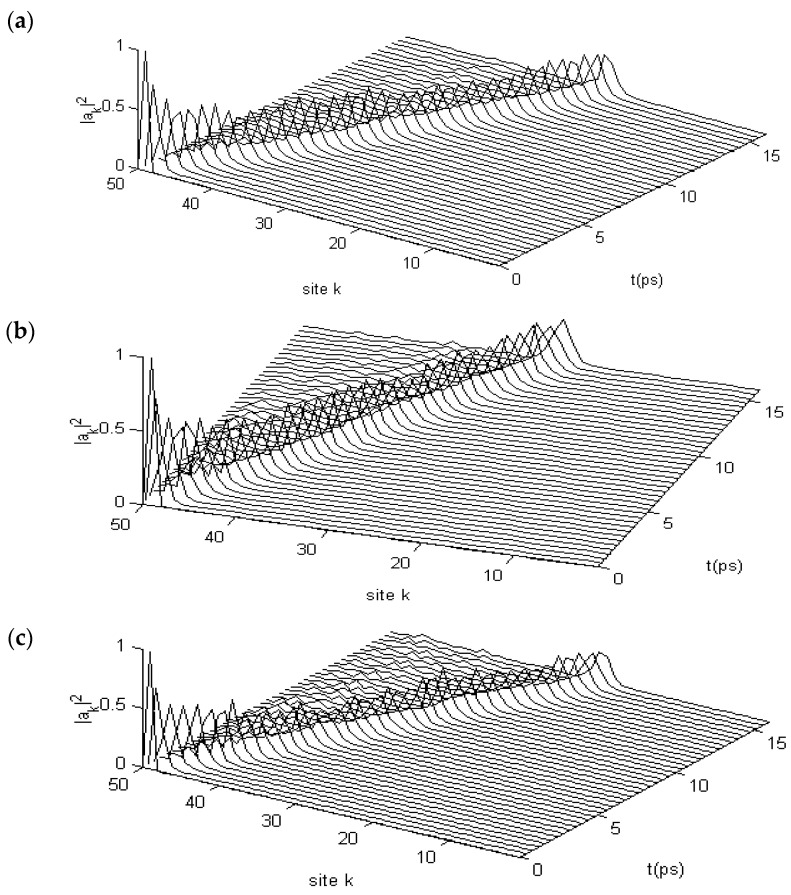

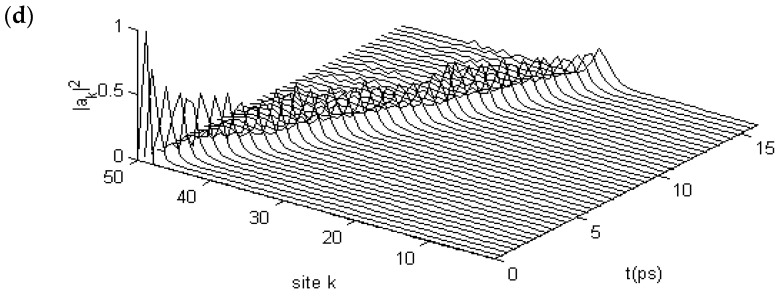
States of Pang’s soliton in the case of (**a**) $\left|\vec{E}\right|$ = 25,500; (**b**) 51,000; (**c**) 76,500; and (**d**) 102,000 V/m, respectively, where “site k” denoted the number of amino acid residue, ”*n*”.

When protein molecules are exposed to EFs, the states of the soliton will change because of variations in the dipole–dipole interaction *J*, which would be replaced by $J+\vec{E}.\vec{p}=J+\left|\vec{E}\right|\left|\vec{p}\right|$ at $\theta =0^{0}$, as shown previously. As such, studies should explore further the properties of various states of the soliton. We studied these variations by using Equations (15)–(19) and the fourth-order Runge–Kutta method [107,108] under the influences of different strengths of EFs with $\left|\vec{E}\right|$ = 25,500, 51,000, 76,500, and 102,000 V/m, which correspond to the variations of the dipole–dipole interaction energy of $\Delta J=J-\bar{J}$ = 5%, 10%, 15%, and 20%, respectively, between the neighboring amino acid residues arising from the EF, in which the electric dipole moment $\left|\vec{p}\right|$ = 10 debyes is used, in which other parameters used by their average values , that are $\bar{M},\bar{w},\bar{\varepsilon_{0}},\bar{\chi_{1}},$ and $\bar{\chi_{2}}$. Figure 4 shows the states of movement of the soliton under these conditions. For instance, the range of variations increases with the increase of strength of the EFs, such that the soliton is stable at $\left|\vec{E}\right|$ = 25,500 and 51,000 V/m because its amplitudes have not been altered during the transport processes. However, the soliton disperses at $\left|\vec{E}\right|$ = 76,500 V/m, as shown in Figure 4c, and disperses significantly at $\left|\vec{E}\right|$ = 102,000 V/m, as shown in Figure 4d. Also, Figure 4 shows that their amplitudes are changed or reduced in these cases and that small ripples occur around the solitons in Figure 4c,d. This occurrence indicates that the states of the soliton, that transport the bio-energy, are changed because of influences by the EFs. This finding suggests that the features of bio-energy are highly sensitive to changes in the dipole–dipole interaction or the states and properties of externally applied EFs.

#### 3.2.2. Results in α-Helix Protein Molecules with Three Channels

We also numerically simulated the dynamic states of the soliton that occurred in the α-helix protein molecules with three channels under the influence of EFs. The Hamiltonian and the wave function of the α-helix protein molecules in Pang’s model [86,87,88,89,90,91,92,93,94,95,96,97,98,99,100,101] are, respectively, represented by

(20) $$\begin{array}{l} H=\sum_{n\alpha }[\varepsilon_{0}B_{n\alpha }^{+}B_{n\alpha }-J(B_{n\alpha }^{+}B_{n+1\alpha }+B_{n\alpha }B_{n+1\alpha }^{+})]+\sum_{n\alpha }[\frac{P_{n\alpha }^{2}}{2M}+\frac{1}{2}w{\left(q_{n\alpha }-q_{n-1\alpha }\right)}^{2}]+\sum [\chi_{1}(q_{n+1\alpha }-q_{n-1\alpha })\times \\ B_{n\alpha }^{+}B_{n\alpha }+\chi_{2}(q_{n+1\alpha }-q_{n-1\alpha })(B_{n+1\alpha }^{+}B_{n\alpha }+B_{n\alpha }^{+}B_{n+1\alpha })+L(B_{n\alpha }^{+}B_{n+1\alpha }+B_{n\alpha }^{+}B_{n-1\alpha })],\alpha =1,2,3. \end{array}$$

and

(21) $$\begin{array}{l} |\Phi (t)\rangle =|\alpha (t)\rangle |\beta (t)\rangle =\frac{1}{\lambda }[1+\sum_{n\alpha }\alpha_{n\alpha }(t)B_{n\alpha }^{+}+\frac{1}{2!}{\left(\sum_{n\alpha }\alpha_{n\alpha }(t)B_{n\alpha }^{+}\right)}^{2}]{|0\rangle }_{ex}\times \\ \exp\{-\frac{i}{\hbar }\sum_{n}[q_{n\alpha }(t)P_{n\alpha }-\pi_{n\alpha }(t)u_{n\alpha }{|0\rangle }_{ph} \end{array}$$

where *L* is the coefficient of the chain–chain interactions among the three channels, and α = 1, 2, 3 denote the three chains. According to this above method, from Equations (20) and (21) we can get the dynamic equations of the soliton in the discrete case, which are

(22) $$\begin{array}{l} i\hbar \dot{\alpha_{n\alpha }(t)}=\varepsilon_{0}\alpha_{n\alpha }(t)-J[(\alpha_{n+1\alpha }(t)++\alpha_{n-1\alpha }(t))]+\chi_{1}[(q_{n+1\alpha }(t)-q_{n-1\alpha }(t)]\alpha_{n\alpha }(t)+\chi_{2}[q_{n+1\alpha }(t)-q_{n-1\alpha }(t)]\times \\ \left[(\alpha_{n+1\alpha }(t)+\alpha_{n-1\alpha }(t)]+\frac{5}{2}\{W(t)-\frac{1}{2}\sum_{m}[q_{n\alpha }(t)\pi_{m\alpha }(t)-{\dot{\pi }}_{n\alpha }(t){\dot{q}}_{m\alpha }(t)\}\alpha_{n\alpha }(t)+L[\alpha_{n\alpha +1}(t)+\alpha_{n\alpha -1}(t)\right] \end{array}$$

(23) $$\begin{array}{l} M\overset{..}{q}\dot{{}_{n\alpha }(t)}=w[q_{n+1\alpha }(t)-2q_{n\alpha }(t)+q_{n-1\alpha }(t)]+2\chi_{1}[{\left|\alpha_{n+1\alpha }(t)\right|}^{2}-{\left|\alpha_{n-1\alpha }(t)\right|}^{2}]+\\ 2\chi_{2}\{\alpha_{n\alpha }^{*}(t)[\alpha_{n+1\alpha }(t)-\alpha_{n-1\alpha }(t)]+\alpha_{n\alpha }(t)[\alpha_{n+1\alpha }^{*}-\alpha_{n-1\alpha }^{*}(t)]\} \end{array}$$

By means of Equations (22) and (23) and the fourth-order Runge–Kutta method [107,108] mentioned previously, we can also numerically simulate the features of the solutions of Equations (22) and (23), which are denoted in Appendix B.

**Figure 5:**
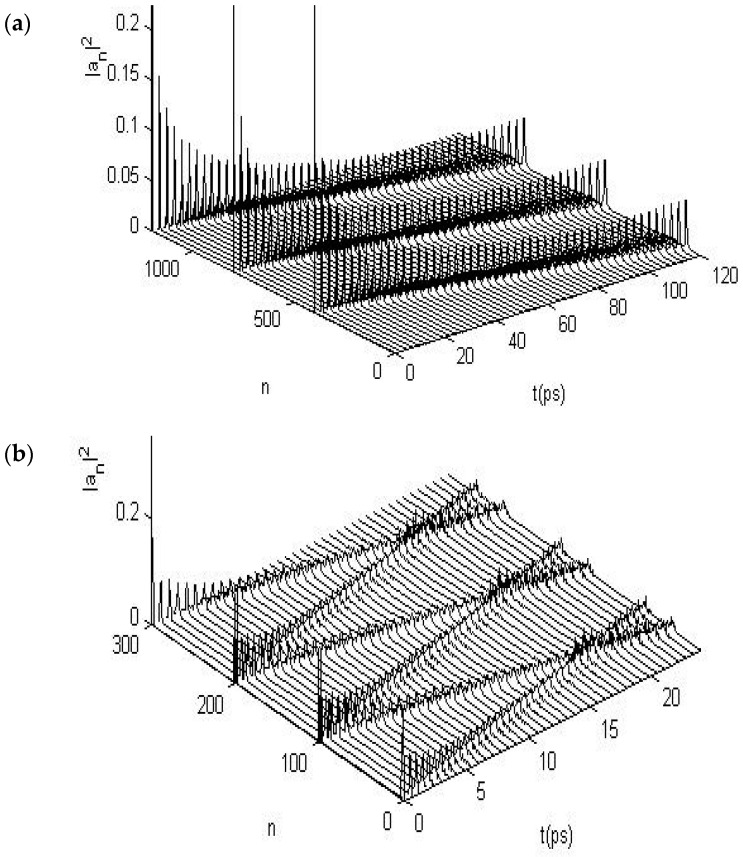
States of (**a**) movement and collision features of (**b**) Pang’s solitons in uniform α-helix proteins.

In this numerical simulation, we use the fourth-order Runge–Kutta method [107,108] with the initial condition of $a_{n\alpha }(0)=A'\sec h[(n\alpha -n_{0}\alpha )\times ({\bar{\chi }}_{1}+{\bar{\chi }}_{2})/4\bar{J}\bar{w}]$, where *A’* is the normalization factor. Meanwhile, we use $\bar{M}$ = 5.73 × 10^−25^ kg, $\bar{w}$ = 39 N/m, $\bar{\varepsilon_{0}}$ = 0.2055 *eV*, $\bar{J}$ = 9.68 × 10^−4^ eV, $\bar{\chi_{1}}$ = 6.2 × 10^−11^ N, $\bar{\chi_{2}}$ = (10 − 18) × 10^−12^ N, and $\bar{L}$= 1.5 meV for the α-helix protein molecules in the simulation, which are widely accepted in energy-transport investigations [24,25,26,27,28,29,64,65,66,67,68,69,70,71,72,73,74,75,76,77,78,79,80,81,82,83,84,85,86,87,88,89,90,91,92,93,94,95,96,97,98,99,100,101]. The numerical solutions of Equations (22) and (23) and their variations for the protein molecules with three channels are shown in Figure 5a, which shows the motion behaviors of the solution, in which the previously given initial conditions are motivated simultaneously on the first ends of the three chains. From this figure, we see that their solution can always retain a bell shape while moving over a long distance of the spacing of 400 amino acid residues in the period of 40 ps along the molecular chains without the dispersion. This feature is similar to the numerical results in Figure 2 for the single-chain proteins. On the basis of the soliton theory [9,27,36], we can also affirm from this result that this solution of Equations (22) and (23) also indicate the existence of a soliton in this case. Therefore, Equations (22) and (23) can be used to simulate the dynamic properties of the soliton excited in the protein molecules with three channels under the action of EF.

We studied the collision property of two solitons, setting up from opposite ends of the three channels in the protein molecules by using the fourth-order Runge–Kutta method. The result is shown in Figure 5b, in which the initial conditions given simultaneously motivate the opposite ends of the three channels, where the initial two solitons separating the 100 amino acid spaces in each channel collide with each other after approximately 17 ps. After this collision, the two solitons in each channel go through each other without scattering to propagate toward and separately along the three chains, which is also similar to the rules of collision for two solitons in the numerical results shown in Figure 3 for single-chain proteins. In accordance with the soliton theory [9,27,34], we can also confirm from the two results in Figure 5 that this solution to Equations (22) and (23) is still a soliton. Thus, by using this method, we can study the changes to the properties of energy transported by the solitons in Equations (22) and (23) under the influences of EFs.

**Figure 6:**
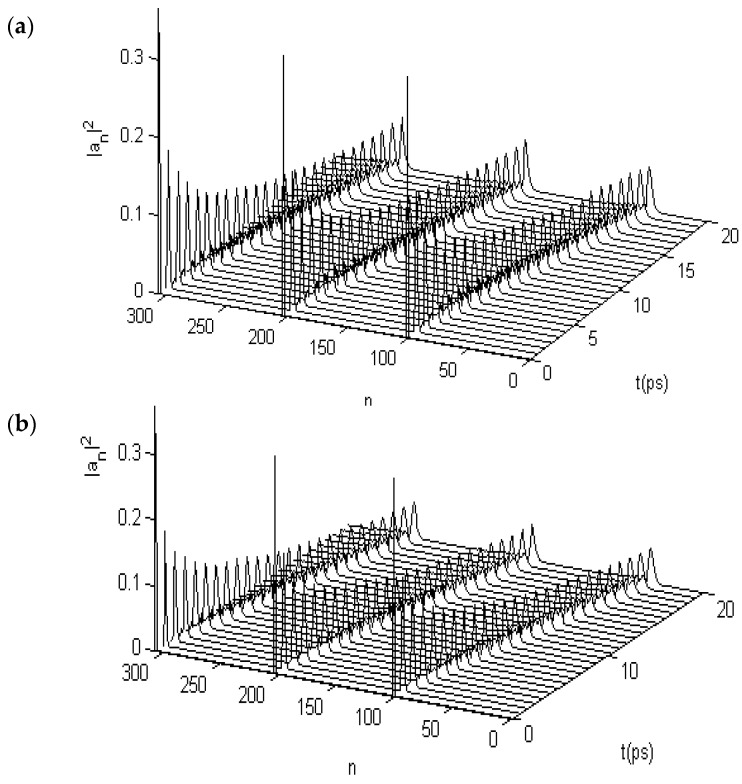

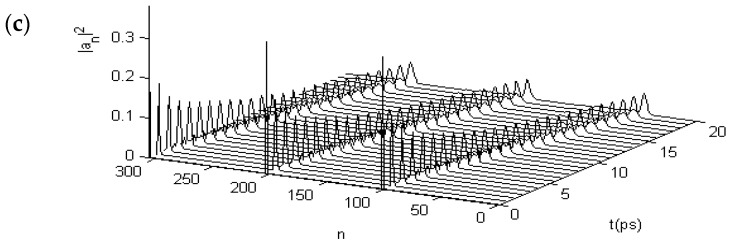
States of solitons in α-helix protein molecules of which $|\vec{E}|$ = (**a**) 17,000; (**b**) 25,500; and (**c**) 34,000 V/m, respectively.

When the protein molecules with the three channels are acted on by an EF, the states of the soliton will also change because of variations in the dipole–dipole interaction *J*, as noted previously. We studied the changing states of movement of the soliton under the effects of EFs of different strengths by using Equations (22) and (23) and the fourth-order Runge–Kutta method [107,108], in which the dipole–dipole interaction *J* is replaced by $J+\vec{E}.\vec{p}=J+\left|\vec{E}\right|\left|\vec{p}\right|$ at $\theta =90^{0}$; where *J* = 9.68 × 10^−4^ eV and $\left|\vec{p}\right|=10$ debyes; other parameters are used as their average values, $\bar{M}$, $\bar{w}$, $\bar{\varepsilon_{0}}$, $\bar{\varepsilon_{0}}$, $\bar{\chi_{1}}$, and $\bar{\chi_{2}}$, as described. Figure 6 shows the states of movement of the solitons in the cases of $\left|\vec{E}\right|$ = 17,000, 25,500, and 34,000 V/m, which correspond to variations of the dipole–dipole interaction energy of $\Delta J=J-\bar{J}$ = 3%, 5%, and 7% between the neighboring amino acid residues, respectively. From these figures, we see clearly that the states of the soliton vary with increasing EF strength. The results show that the soliton is stable at $\left|\vec{E}\right|$= 17,000 V/m but that it disperses at $\left|\vec{E}\right|$ = 25,500 V/m and damps at 34,000 V/m. The stability of solitons in three-channel α-helix protein molecules is reduced with respect to the solitons in single-chain protein molecules under the influence of an EF through the existence of the disperse effect, which is caused by the chain–chain interactions among the three channels. Thus, we can surmise that the state of a soliton transporting energy is highly sensitive to the EFs in EMFs.

Marracino et al. [112] showed the effects of EFs in EMFs on chemical reactions and determined that a 100,000 V/m electric field can have a significant effect on chemical reaction in micelles. In such a case, we think that this result might serve somewhat as a basis of comparison. These comparisons indicate the relevance of our investigations of the biological effects of EFs or EMFs because the strengths of electric field, as described previously, can generate corresponding electrically interactional energies to the amino acid residues within a few nanometers, which is approximately one order of magnitude smaller than that of the dipole–dipole interactional energy J between them. Therefore, these estimated values are basically the same as those in the calculations previously described, which, we believe, are basically correct.

## 4. Experimental Evidences for this Theory

The theoretical results and mechanisms of the biological effects of EMFs mentioned previously require further experimental confirmation. Experimental evidence should relate mainly to the affirmation of the nonlinear excitation or the soliton, which provides the bio-energy transport in an α-helix protein molecule, and its changes with varying EF in EMF. Investigations indicate that the nonlinear interaction, or the soliton, can be confirmed from the infrared spectrum of absorption from the α-helix protein molecule [113]. The essence of the experimental evidence is to confirm the real existence of the soliton in transporting the bio-energy within the α-helix protein molecules. Therefore, the first step of the investigation is to determine the existence of soliton excitation in the protein molecules with α-helix structures. Subsequently, investigation should focus on the exact influence of the EF on the soliton. By confirming the existence of a soliton and indicating that EMFs can influence or change the properties of the soliton, we can confirm the correctness of the previously given mechanics for the biological effects of EFs, that is, EMFs can influence the bio-energy transport in protein molecules. Careri et al. [113,114,115,116,117], Scott [30,31,118,119,120,121], and Alexander et al. [122,123] measured the infrared spectrum of absorption for acetanilide (ACN) [113], a molecular structure of like-protein molecules. From these investigations, they confirmed that the 1666- and 1650-cm^−1^ bands that occurred in the infrared spectra represent the excited states of amide-I mode in ACN, which corresponds to the excitations of the exciton and the soliton, respectively. Their infrared absorption strengths change linearly and exponentially by increasing the temperatures in ACN, respectively. Thus, the existence of the soliton in ACN was affirmed [115,116,117,118,119]. We chose a collagen protein with α-helix conformation to confirm the existence of the soliton and study the influence of EFs, and to affirm further the validity of the mechanism and theory previously described. These investigations are summarized as follows.

### 4.1. Experimental Evidence of the Existence of Solitons in Protein Molecules

**Figure 7:**
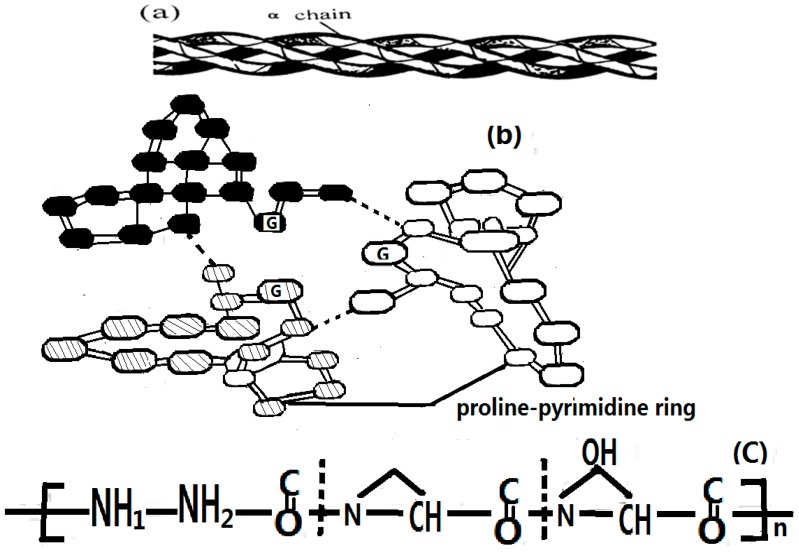
Molecular structure of collagen: (**a**) fundamental structure of right α-helix with three channels; (**b**) atomic distribution on top section of three channel–axes, where G is carbon atom, is hydrogen bond; (**c**) one-dimensional structure of gly-pro-hydropro.

Because collagen is a common biomacromolecule that widely exists in a solid-like or soft condensed state from 0 to 95 °C in living systems, it is a basic component of surface-tissue musculature, e.g., smooth muscle [113,114,115,116,117,118,119,120,121,122,123,124,125]. Tropocollagen, a kind of collagen, is an α-superhelical biopolymer with three channels (Figure 7), in which each α-peptide chain contains 1050 amino acid residues and a sugary side chain. The structure consists of approximately 35% glycine, 10% proline, and 9% hydroproline, as well as some alumine and hydrolysin. Its molecular structure might be described as follows. Its primary conformation is a chain of (Gly-X-Y)_n_, where X and Y are the proline and the hydroproline, respectively. Its secondary structure consists of main chains that are regularly folded into an α-helical chain with a left-hand spin, in which the glycine is at the center of the helix and the proline and hydroxylproline are at its exterior. If the three chains are again folded into a multi-helix structure, a tertiary conformation is constructed in which each chain contains several helices. When the three chains are assembled and wind further into a right-hand spin to construct a long fiber, in virtue of the hydrogen bonds and the C=O…NH groups, its quaternary conformation is obtained. Thus, the linkage between the two lysine residue side groups in the peptide chains occurs through covalent bonding, which is formed and produced by the oxidoreductase reaction of lysine, as represented by –CO–CH(NH)–CH_2_–CH_2_–CH_2_–CH=N–CH_2_–CH_2_–CH_2_–CH_2_–CH(NH)–CO–. Collagen is, therefore, a kind of α-helical protein molecule with an α-helix conformation (Figure 7), in which the hydrogen bonds and the covalent bonds are arranged alternately, which plays an important role in stabilizing the molecular structure of the collagen and completing its biological functions (such as the transport of energy and information) and enhancing its tensile strength. We used the features of the infrared spectrum to confirm the existence of the soliton and the influences of EMF and could thus verify further the validity of the previously given mechanism and theory.

We measured the infrared spectrum of absorption of the collagen, which was supplied by Sigma-Aldrich (St. Louis, MO, USA), using the spectrum GXFFIR spectrometer with a DTGS detector and a variable-temperature bath with a reported accuracy of ±1 °C, in which infrared silicon–carbon bars served as a light source, was provided by Perkin Elmer (Waltham, MA, USA), and a 670 Nicolet FT-IR spectrometer with a 4-cm^−1^ resolution and ATR full-reflective device with 16 successive scans were supplied by Nicolet Nexus (Ramsey, MN, USA), respectively. In this experiment we measured and collected mainly the infrared absorption spectrum of the collagen in the region of 1000–2000 cm^−1^ by using, first, the Perkin Elmer spectrum GXFFIR spectrometer [108,109,110,111,112,113,114,115,116,117,118,119,120,121,124,125,126] and, again, the 670 Nicolet FT-IR and the GXFFIR spectrometers with resolutions of 4 cm^−1^ to check the results. The measured samples of collagen were sandwiched between KBr windows at temperatures controlled by a variable-temperature bath. Throughout the experiment, we inspected the infrared absorption spectrum of the collagen in the range of 1000–4000 cm^−1^. To obtain an acceptable signal-to-noise ratio, we took 16 scans of these infrared spectra. When the temperatures of the samples changed from 15 to 95 °C, variations of the infrared spectra of the collagen were also measured and inspected by a spectrometer. In this experiment, the water and the water vapor in these samples were completely drained from the tested samples so as not to influence results.

**Figure 8:**
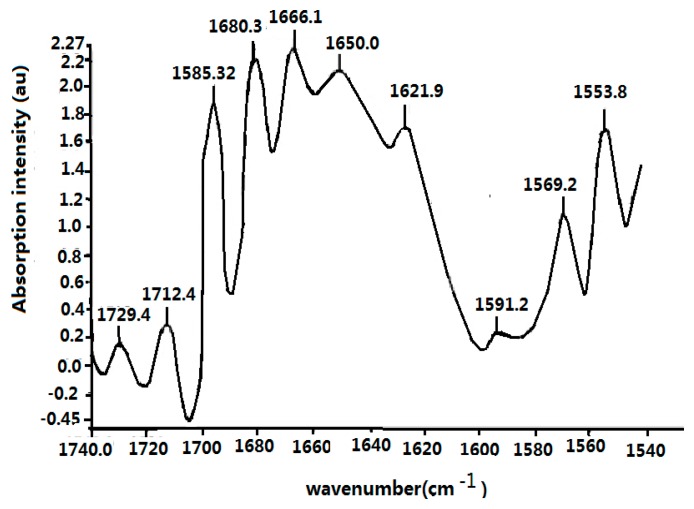
The infrared spectrum of collagen in 1540–1740 cm^−1^.

The infrared spectrum of collagen at 25 °C in the range of 1540–1710 cm^−1^ is shown in Figure 8. Its spectrum at high frequencies is dominated by the amide spectrum, in which three clear Amide I vibrational modes are present: 1666.11, 1680.38, and 1695.32 cm^−1^, where an important feature in the spectrum is the appearance of a new band at 1650.01 cm^−1^. Other amide bands, such as the Amide II at 1624.9 cm^−1^, the Amide III at 1553.1, and so on are also observed and obtained from the previously designated.

The infrared spectrum of collagen denoted in Figure 8 has the following properties: (1) Amide II, III bands, in addition to an Amide I band, exist in the infrared absorption spectrum of the collagen; (2) a new band of 1650 cm^−1^ appears besides the conventional 1666 cm^−1^ band. These results are very interesting and important because from this experiment we confirm the appearances of 1650 cm^−1^ and 1666 cm^−1^,which correspond the excitations of soliton and exciton in the collagen in this case, respectively, in accordance with Careri et al. [113,114,115,116,117] and Scott [30,31,118,119,120,121] researched conclusions. Thus we may use the method and material to investigate further the influences of EF in EMF on the soliton, or energy transport in collagen, which is described in detail as follows.

**Figure 9:**
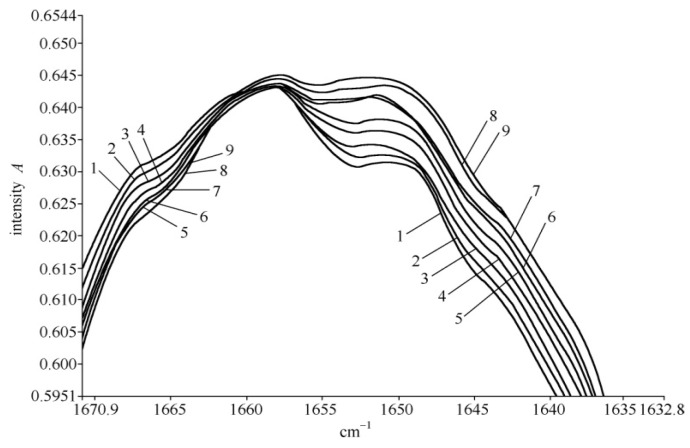
Changes of intensity for infrared absorption of collagen in the region at (1) 95 °C; (2) 85 °C; (3) 75; (4) 65 °C; (5) 55 °C; (6) 45 °C; (7) 35 °C; (8) 25 °C; and (9) 15 °C.

At the same time, the infrared spectra of absorption of collagen at different temperatures are carefully recorded, where the temperatures of the samples varied from 15 to 95 °C, with the intervals of 10 °C and a reported accuracy of ±1 °C, which is controlled by variable-temperature equipment. The temperature-dependent intensity of infrared absorption at temperatures ranging from 15 to 95 °C is shown in Figure 9. Figure 9 clearly indicates that the intensity of the 1650-cm^−1^ band increases with the decrease of temperature, without any apparent change in frequency and shape, but is weakened at 95 °C. However, the strength of the Amide I infrared absorption of the 1666-cm^−1^ band decreases with a decrease in temperature.

The peak strengths of the 1650- and 1666-cm^−1^ bands versus temperature are clearly shown in Figure 9. Although the temperature dependences of strengths for the 1650- and 1666-cm^−1^ bands differ, the absorption strength of the 1666-cm^−1^ band increases linearly with increasing temperature; however, the strength of the 1650-cm^−1^ band decreases exponentially with an increase in temperature This relationship can be approximately simulated in the exponential form of

**Figure 10:**
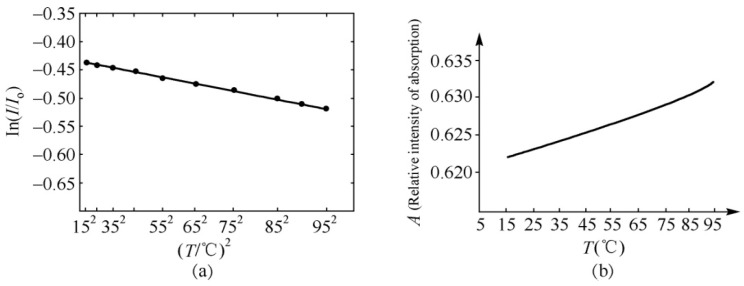
Temperature dependences of intensity of peaks in the infrared spectrum of collagen: (**a**) Relation of logarithm of relative intensity, Ln (*I*/*I*_0_), versus (T/°C)^2^ for a 1650-cm^−1^ peak, where “●” denotes experimental data; and (**b**) linear temperature dependence of relative intensity, *I*/*I*_0_, for a 1666-cm^−1^ peak.

*I* = *I*_0_ exp {−[0.437 + 8.987 × 10^−6^ (T/°C)^2^]} (24)

where T is represented in Celsius temperature (°C); and *I*_0_ is a constant related to the initial strength. Figure 10 shows the experimental data of the relationship between the logarithm of relative strength, Ln (*I*/*I*_0_), and the temperature, (T/°C)^2^, for the 1650-cm^−1^ peak in the collagen, and this relationship can be simulated by Ln (*I*/*I*_0_) = −(0.437 + 8.987 × 10^−6^ (T/°C)^2^). It denotes that the strengths of the 1650-cm^−1^ peak in the infrared spectrum of absorption in the collagen decreased exponentially with increasing temperatures. Figure 10b gives the linear changed relation of relative strength, *I*/*I*_0_, for the 1666-cm^−1^ peak changing with increasing temperatures in the infrared spectrum of the absorption of the collagen molecule, a novel and interesting finding.

On the basis of the experimental results in Figure 8, Figure 9 and Figure 10, we can affirm again the existence of solitons in collagen according to the theory and conclusions of Careri et al. [113,114,115,116,117], Scott [30,31,108,109,110,111,112,113,114,115,116,117,118,119,120,121], and Alexander et al. [122,123]. Taking into account these results, we studied further the influences of EMFs on solitons, because the outline and features of solitons depend closely on the dipole–dipole interaction between the neighboring amino acid residues with certain electric dipole moments in the collagen, as mentioned previously.

### 4.2. Experimental Evidence of the Influence of EFs on Solitons in Protein Molecules

We investigated and measured further the influence of EMFs on the properties of the 1650-cm^−1^ peak in collagen, which is related to the soliton excited, by using the 670 Nicolet FT-IR spectrometer based on the above results. If we obtain the changes of features of the peak of 1650 cm^−1^ by varying the externally applied electric voltage, we can affirm that the EF varies the states and features of the soliton, or the bio-energy transport, in the collagen because of variations of the electric dipole moments of amino acids in the protein.

**Figure 11:**
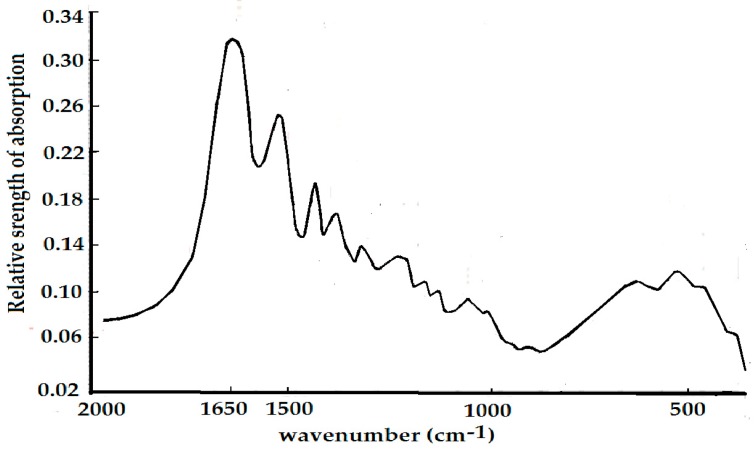
Infrared spectrum of absorption of collagen in 480–2000 cm^−1^.

**Figure 12:**
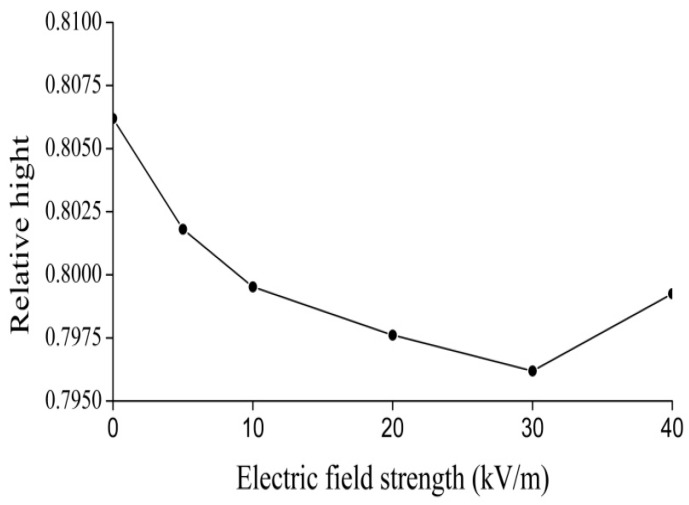
Changes in the relative height of a 1650-cm^−1^ peak with increases to externally applied voltage.

**Figure 13:**
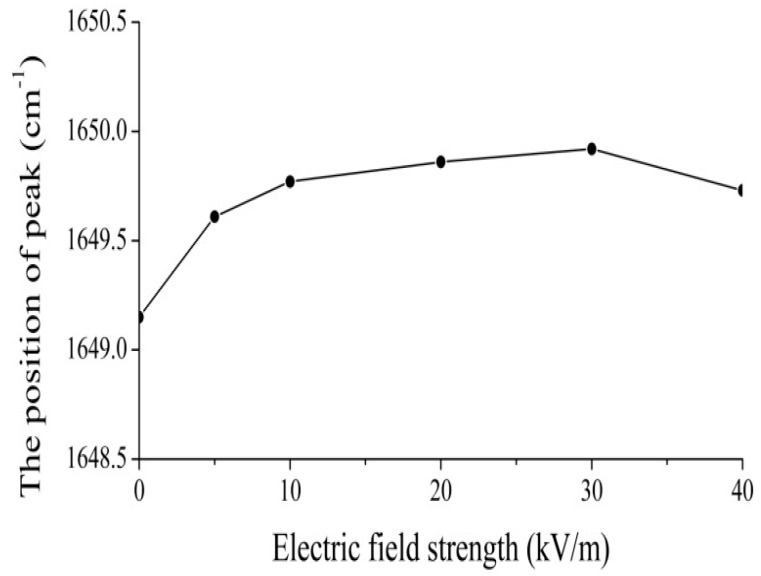
Changes in the position of a 1650-cm^−1^ peak with increases to the externally applied voltage.

**Figure 14:**
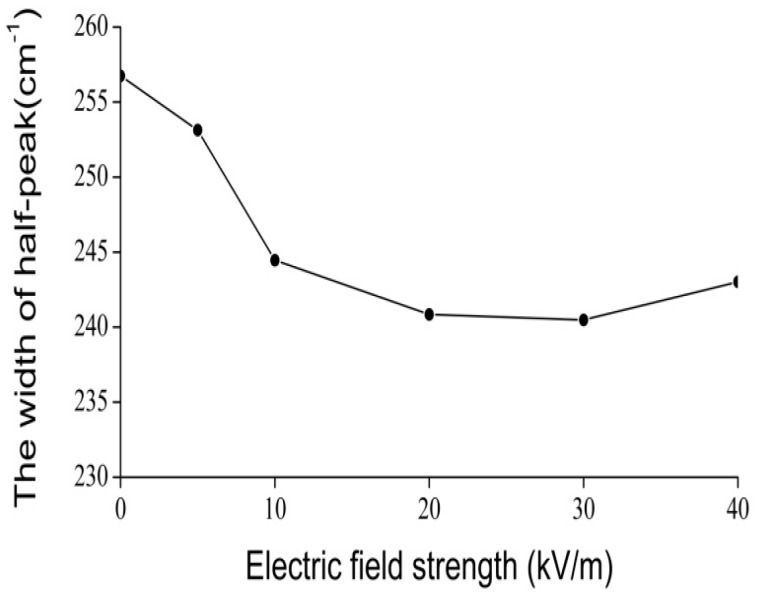
Changes to the width of a half-peak for a 1650-cm^−1^ peak with increases to the externally applied voltage.

In this experiment the electric voltage, which is applied on the collagen, changed from 15,000 to 20,000 V, and our results are shown in Figure 11, Figure 12, Figure 13 and Figure 14. Figure 11 shows the results of the infrared spectrum of absorption of collagen in 480–2000 cm^−1^, and it is apparent that the 1650 cm^−1^ peak occurs in this case. Hence, the soliton exists within the collagen in this case. In this experiment, the externally applied voltages are linked on the two sides of the thin plates, where the measured collagen samples are first made into powders, which are again inserted into the center of the thin plates and were kept in tight contact with each other through extrusion and pressurization. Subsequently, they were placed in the sample bath in the FT-IR spectrometer. When the electric circuit built was connected, the collagen samples were exposed to the externally applied EF. We could then measure and record the infrared spectra of the collagen samples and their variations (Figure 12, Figure 13 and Figure 14). Figure 12 shows the decreases in the peak height with increases in EF strength up to 30,000 V/m, and the inverse beyond 30,000 V/m. Figure 13 and Figure 14 indicate changes in the position of the 1650-cm^−1^ peak and its half-peak width with increases to the externally applied EF strength. Figure 13 clearly shows that the position of this peak lifts with increases to the EF strength below 30,000 V/m but lowers sluggishly after 30,000 V/m. For its half-peak width, we find that the half-peak width decreases with increases to the EF strength up to 30,000 V/m, but it increases sluggishly after 30,000 V/m (Figure 14). Therefore, Figure 12, Figure 13 and Figure 14 exhibit clearly that the features of the 1650-cm^−1^ peak vary under the influences of externally applied electric voltages, which are basically consistent with those in Figure 6. Thus, we can affirm and judge from these experiments that the externally applied EFs influence and change the states and features of the soliton and the bio-energy transport in the collagen (Figure 12, Figure 13 and Figure 14). Hence, we verified the validity of these results and the mechanism of influence of EMFs and EFs on life bodies through changes of the bio-energy transport or in the features of the soliton caused by changes to the electric properties of amino acids in protein molecules.

## 5. Conclusions

The mechanism of influence of an externally applied EF or EMF on bio-energy transport along a protein molecule and their properties are investigated by using analytic methods and numerical simulation as well as experimental measurements. Energy, released by the hydrolysis reaction of an ATP molecule, and its transport are the basic biological activities in the life system, including muscle contraction, DNA duplication, neuroelectric pulse transfer along the neurolemma, and calcium and sodium pumping. This transport is conducted through movement of the soliton along the protein molecules and the dipole–dipole interaction between neighbor amino acid residues. Thus, we affirmed that externally applied EFs or EMFs can directly change the strength and direction of electric dipole moments of amino acid residues in protein molecules, which results directly in variations of property of the bio-energy transported by the soliton, thus a series of new biological effects can occur in living systems. In this investigation we obtained the following results.

The first result confirms one mechanism of the biological effect of EMFs and EFs, that is, the EFs and EMFs changed the features of bio energy transport through variations of electric dipole moments of the amino acid residue or the dipole–dipole interactional energy between the neighboring amino acid residues. Thus, we determined first that the targets of the biological effects of EMFs and EFs are the amino acid residues in protein molecules.

The second result establishes a theory of the biological effects of EMFs and EFs through the study of soliton mechanisms and bio-energy transport in protein molecules. In this theory, the dipole–dipole interactional energy J between neighboring amino acid residues is replaced by $J+\vec{E}.\vec{p}=J+\left|\vec{E}\right|\left|\vec{p}\right|\cos\theta$. We found changes to the features of bio-energy transport in the Pang’s soliton under different EFs using analytical, numerical, and experimental methods.

Our analytical investigations indicated that EMFs and EFs influenced both the amplitude of the soliton as well as its form and outline. Its degree of effect depended on the strength of the EF, as well as its direction with respect to the electric dipole moments of amino acid residues. Thus, the biological effects of EFs and EMFs decrease with increases to the angle *θ* between them. If $\theta =0^{0}$, then $\vec{E}.\vec{p}=\left|\vec{E}\right|\left|\vec{p}\right|$, but $\vec{E}.\vec{p}=0$ at $\theta =90^{0}$, therefore, EMFs and EFs have no biological effect in this case, even at high strengths. Hence, different EMFs and EFs have different biological effects as well, whereas the same EMFs and EFs also have different biological effects because their effects are determined by both the strength and the direction of the EMF or the EF. However, this effect has obvious randomness at the macroscopic level.

In the numerical simulation, we found that EMFs and EFs change the states and features of the movement of the soliton by using the fourth-order Runge–Kutta numerical simulation method in Pang’s model [89,90,91,92,93,94,95,96,97,98,99,100,101,102,103,104,105]. By applying this simulation, we found that the solitons are stable at EF strengths of 25,500 and 51,000 V/m, but the solitons disperse at 76,500 V/m and significantly so at 102,000 V/m for protein molecules with single chains. However, the soliton is stable only at the EF strength of 17,000 V/m. It disperses at 25,500 V/m and damps at 34,000 V/m in protein molecules with three channels. This finding implies that the stability of a soliton in a three-channel α-helix protein molecule is reduced compared with that in a single-chain protein molecule because of the influence of the dispersed effect of chain–chain interactions among the three channels under the actions of EFs and EMFs. At the same time, we found that the capability of the bio-energy transported by the soliton is depressed considerably if the strength of the EMF or the EF is very high. These results verified that EMFs and EFs can vary the properties of the energy transport and the states of the soliton and that other biological effects could occur in living systems.

Finally we inspected and confirmed experimentally the real existence of the soliton, which corresponds to the excitation of a 1650-cm^−1^ peak, by using the infrared spectra of absorption of collagen with an α-helical structure and its change of features with varying external EFs, in which the height, position, and half-peak width of the soliton vary with different external EFs.

However, we should note the following problems associated with these investigations.

(1) We have not studied concretely the macroscopic biological effects arising from changes to energy transport under the influences of EMFs and EFs. Thus, further research into the molecular and cellular biology must be pursued. Thus, we cannot confirm that the concrete biological effects arising from variations in the bio-energy transport in protein molecules in the presence of EMFs or EFs are advantageous or harmful to the health of humans and animals.
(2) The mechanism of the biological effects of EFs and EMFs that we propose require additional experimental confirmation, i.e., through instrumentation and novel methods, taking direct measurements of any changed features in the electric dipole moments of α-helix proteins or of the amino acid residues in them arising from EMFs and EFs, which entails many challenges.
(3) Because the strength of EMF frequency is altered in a real EMF, we studied the biological effects of static EFs or DC fields only. Therefore, further investigations would focus on the biological effects of AC fields or altered EFs.

## Figures and Tables

**Table 1:** Comparison of soliton features in Pang’s model and the Davydov model.

	Lifetime at 300 K (S)	Critical Temperature (K)	Number of Amino Acids Traveled by Soliton In Lifetime	Nonlinear Interaction *G* (×10^−21^ J)	Amplitude of Soliton	Width of the Soliton (×10^−10^ m)	Binding Energy of Soliton (×10^−21^ J)
Pang’s model	10^−9^–10^−10^	320	Several hundreds	1.18	0.974	14.88	−0.188
Davydov model	10^−12^–10^−13^	<200	<10	3.8	1.72	4.95	−7.8

## Appendix A. Solutions to Equations (17)–(20)

Note that these are base equations derived from numerical computer simulations and the Runge–Kutta method.

(A1) $$\begin{array}{l} \alpha r_{j,n+1}=\alpha r_{jn}+\frac{h}{6}K1_{j}+2K2_{j})+3K3_{j}+K4_{j}),\\ \alpha i_{j,n+1}=\alpha i_{jn}+\frac{h}{6}(L1_{j}+2L2_{j}+2L3_{j}+L4_{j}),\\ q_{j,n+1}=q_{jn}+\frac{h}{6}(M1+2M2_{j}+2M3_{j}+M4_{j}),\\ q_{n+1}-q_{n-1})\alpha i_{n}+\chi_{2}(q_{n+1}-q_{n})(ai_{n+1}+\alpha i_{n-1}),\\ y_{j,n+1}=y_{jn}+\frac{h}{6}(N1_{j}+2N3_{j}+N4_{j}), \end{array}$$

where

(A2) $$\begin{array}{l} K1=-\frac{J}{\hbar }(\alpha i_{j+1}+\alpha i_{j-1n}+\frac{\chi_{1}}{\hbar }(q_{j+1n}-q_{j-1n})\alpha i+\frac{\chi_{2}}{\hbar }(q_{j+1n}-q_{jn})(\alpha i_{j+1n}-\alpha i_{j-1n}),\\ K2_{j}=K1_{j}-\frac{Jh}{2\hbar }(L1_{j}+2L2_{j}+2L3_{j}+L4_{j})+\\ \frac{\chi_{1}h}{2\hbar }(M1_{j+1}-M1_{j-1})(ai_{jn}+\frac{h}{2}L1_{j})+\frac{\chi_{2}h}{2\hbar }(q_{j+1,n}-q_{jn})(L1_{j+1}+L1_{j-1}),\\ M1_{j}=y_{jn}/M;\\ M2_{j}=M1_{j}+\frac{h}{2M}N1_{j};\\ M3_{j}=M1_{j}+\frac{h}{2M}N2_{j};\\ M4_{j}=M1_{j}+\frac{h}{M}N3_{j};\\ N1_{j}=W(q_{j+1,n}-2q_{jn}+q_{j-1,n})+2\chi_{1}(ar_{j+1,n}^{2}+ai_{j+1,n}^{2}-ar_{j-1,n}^{2}-ai_{j-1,n}^{2})\\ +4\chi_{2}[ar_{jn}(ar_{j+1,n}-ar_{j-1,n})+ai_{jn}(ai_{j+1,n}-ai_{j-1,n})];\\ N2_{j}=W[q_{j+1,n}-2q_{jn}+q_{j-1,n}+\frac{h}{2}(M1_{j+1}-2M1_{j}+M1_{j-1})]\\ +2\chi_{1}[{\left(ar_{j+1,n}+\frac{h}{2}K1_{j+1}\right)}^{2}+{\left(ai_{j+1,n}+\frac{h}{2}L1_{j+1}\right)}^{2}-{\left(ar_{j-1,n}+\frac{h}{2}K1_{j-1}\right)}^{2}\\ -{\left(ai_{j-1,n}+\frac{h}{2}L1_{j-1}\right)}^{2}]+4\chi_{2}\{(ar_{jn}+\frac{h}{2}K1_{j})[ar_{j+1,n}-ar_{j-1,n}+\frac{h}{2}(K1_{j+1}-K1_{j-1})]\\ +(ai_{jn}+\frac{h}{2}L1_{j})[ai_{j+1,n}-ai_{j-1,n}+\frac{h}{2}(L1_{j+1}-L1_{j-1})]\};\\ N3_{j}=W[q_{j+1,n}-2q_{jn}+q_{j-1,n}+\frac{h}{2}(M2_{j+1}-2M2_{j}+M2_{j-1})]\\ +2\chi_{1}[{\left(ar_{j+1,n}+\frac{h}{2}K2_{j+1}\right)}^{2}+{\left(ai_{j+1,n}+\frac{h}{2}L2_{j+1}\right)}^{2}-{\left(ar_{j-1,n}+\frac{h}{2}K2_{j-1}\right)}^{2}\\ -{\left(ai_{j-1,n}+\frac{h}{2}L2_{j-1}\right)}^{2}]+4\chi_{2}\{(ar_{jn}+\frac{h}{2}K2_{j})[ar_{j+1,n}-ar_{-1,n}+\frac{h}{2}(K2_{j+1}-K2_{j-1})]\\ +(ai_{jn}+\frac{h}{2}L2_{j})[ai_{j+1,n}-ai_{j-1,n}+\frac{h}{2}(L2_{j+1}-L2_{j-1})]\};\\ N4_{j}=W[q_{j+1,n}-2q_{jn}+q_{j-1,n}+h(M3_{j+1}-2M3_{j}+M3_{j-1})]\\ +2\chi_{1}[{\left(ar_{j+1,n}+hK3_{j+1}\right)}^{2}+{\left(ai_{j+1,n}+hL3_{j+1}\right)}^{2}-{\left(ar_{j-1,n}+hK3_{j-1}\right)}^{2}\\ -{\left(ai_{j-1,n}-hL3_{j-1}\right)}^{2}]+4\chi_{2}\{(ar_{jn}+hK3_{j})[ar_{j+1,n}-ar_{j-1,n}+h(K3_{j+1}-K3_{j-1})]\\ +(ai_{jn}+hL3_{j})[ai_{j+1,n}-ai_{j-1,n}+h(L3_{j+1}-L3_{j-1})]\} \end{array}$$

## Appendix B. Solutions to Equations (17)–(20)

From the time-dependent Schrödinger equation

(B1) $$H|\Phi >=i\hbar \frac{\partial }{\partial t}|\Phi >$$

with the Hamiltonian Equation (3) and the time-dependent wave function Equation (4) and

(B2) $$i\hbar \;\frac{\partial }{\partial t}<\left|\;\Phi (t)\right|u_{n}|\Phi (t)>=<\Phi (t)\left|\left[u_{n},H\right]\;\right|\Phi (t)>$$

(B3) $$i\hbar \;\frac{\partial }{\partial t}<\Phi (t)\left|P_{n}\right|\Phi (t)=<\Phi (t)\left|\;\left[P_{n},H\right]\;\right|\;\Phi (t)>$$

and considering further the neighboring interactions among the three channels we can determine

(B4) $$\begin{array}{ll} i\hbar {\dot{a}}_{n\alpha }(t) & =\varepsilon_{0}a_{n\alpha }(t)-J[a_{n+1\alpha }(t)+a_{n-1\alpha }(t)]+\chi_{1}[q_{n+1\alpha }(t)-q_{n-1\alpha }(t)]a_{n\alpha }(t)\\ & \;+\chi_{2}[q_{n+1\alpha }(t)-q_{n-1\alpha }(t)][a_{n+1\alpha }(t)+a_{n-1\alpha }(t)]\\ & \;+\frac{5}{2}\{w(t)-\frac{1}{2}\sum_{m}\left[q_{m\alpha }(t)\pi_{m\alpha }(t)-{\dot{\pi }}_{m\alpha }(t){\dot{q}}_{m\alpha }(t)\right]\}a_{n\alpha }(t)+L[a_{n\alpha +1}(t)+a_{n\alpha -1}(t)] \end{array}$$

(B5) $$\begin{array}{ll} M{\ddot{q}}_{n\alpha } & =W[q_{n+1\alpha }(t)-2q_{n\alpha }(t)+q_{n-1\alpha }(t)]+2\chi_{1}[|a_{n+1\alpha }(t){|}^{2}-|a_{n-1\alpha }(t){|}^{2}]\\ & \;+2\chi_{2}\{a_{n\alpha }^{*}(t)[a_{n+1\alpha }(t)-a_{n-1\alpha }(t)]+a_{n\alpha }(t)[a^{*}{}_{n+1\alpha }(t)-a^{*}{}_{n-1\alpha }(t)]\}. \end{array}$$

Also, we can eliminate the term containing *ε*_0_ in Equation (B4) through the following transformation:

(B6) $$\varphi_{n\alpha }(t)=a_{n\alpha }(t)\exp[-i\varepsilon_{0}t/\hbar ]$$

Because *a_n_*(*t*) in Equations (B4) and (B5) is a complex function, we can make the following transformation

(B7) $$a_{n\alpha }(t)=ar_{n\alpha }(t)+iai_{n\alpha }(t)\text{ with }{\left|a_{n}\right|}^{2}={\left|ar_{n}\right|}^{2}+{\left|ai_{n}\right|}^{2}$$

Thus Equations (B4) and (B5) change as

(B8) $$\begin{array}{ll} \hbar \dot{a}r_{n\alpha } & =-J(ai_{n+1\alpha }+ai_{n-1\alpha })+\chi_{1}(q_{n+1\alpha }-q_{n-1\alpha })ai_{n\alpha }+\chi_{2}(q_{n+1}-q_{n-1\alpha })(ai_{n+1\alpha }+ai_{n-1\alpha })\\ & \;+L[ai_{n\alpha +1}(t)+ai_{n\alpha -1}(t)] \end{array}$$

(B9) $$\begin{array}{ll} -\hbar \dot{a}i_{n\alpha }= & -J(ar_{n+1\alpha }+ar_{n-1\alpha })+\chi_{1}(q_{n+1\alpha }-q_{n-1\alpha })ar_{n\alpha }\\ & +\chi_{2}(q_{n+1\alpha }-q_{n-1\alpha })(ar_{n+1\alpha }+ar_{n-1\alpha })+L[ai_{n\alpha +1}(t)+ai_{n\alpha -1}(t)] \end{array}$$

(B10) $${\dot{q}}_{n\alpha }=\frac{y_{n\alpha }}{M}$$

(B11) $$\begin{array}{ll} {\dot{y}}_{n\alpha }= & W[q_{n+1\alpha }-2q_{n\alpha }+q_{n-1\alpha }]+2\chi_{1}[ar_{n+1\alpha }^{2}+ai_{n+1\alpha }^{2}-ai_{n-1\alpha }^{2}-ai_{n-1\alpha }^{2}]\\ & \;+4\chi_{2}[ar_{n\alpha }(ar_{n+1\alpha }-ar_{n-1\alpha })+ai_{n\alpha }(ai_{n+1\alpha }-ai_{n-1\alpha })] \end{array},$$

where *ar_n_* and *ai_n_* are real and imaginary parts of *a_n_*(*t*), respectively. Therefore, Equations (B8)–(B11) are dynamic in Equations (17)–(20), which can be used to simulate and calculate the dynamic properties of particles. Therefore, the dynamic properties of the peptide groups or amino acid molecules mentioned previously can be calculated and studied through Equations (B8)–(B11), as detailed in Appendix A for single-chain protein molecules.

## References

[1] Pang X.F. (2009). Bio-Electromagnetism.

[2] Fröhlich H. (1988). Biological Coherence and Response to External Stimuli.

[3] Ahlbom A., Day N., Feychting M., Skinner R.E., Dockerty J., Linet J., McBride M., Michaelis M., Olsen J., Tynes T. (2000). A pooled analysis of magnetic fields and childhood leukaemia. Br. J. Cancer.

[4] Dowson D.I., Lewith G., Campbell M., Mullee M.A., Brewster L.A. (1988). Overhead high-voltage cables and recurrent headache and depressions. Practitioner.

[5] Greenland S., Sheppard A.R., Kaune W.T., Poole C., Kelsh M.A. (2000). A Pooled analysis of magnetic fields, wire codes, and childhood leukemia. Epidemiology.

[6] Perry S., Pearl L., Binns R. (1989). Power frequency magnetic field: Depressive Illness and myocardial infarction. Public Health.

[7] Perry F.S., Reichmanis M., Marino A.A., Becker R.O. (1981). Environmental power-frequency magnetic fields and suicide. Health Phys..

[8] Bromberg J., Darnell J.E. (2000). The role of STATs in transcriptional control and their impact on cellular function. Oncogene.

[9] Pang X.F. (2008). Biophysics.

[10] Li G., Pang X.F. (2011). Biological effects of environmental electromagnetic fields. Adv. Mater. Res..

[11] Li G., Pang X.F. (2011). The influence of electromagnetic field on the electromagnetic properties of biological tissue. Prog. Biochem. Biophys. (Chin.).

[12] Li G., Li B., Yang S.F., Pang X.F. (2012). The influences of static magnetic field on the electric features of testicular tissue of rats. Aerosp. Med. Med. Eng..

[13] Li G., Yan Y.Y., Han S.G., Pang X.F. (2012). The influences of static magnetic field on the brain tissue of the rats using infrared spectrum metho. Laser Infrared.

[14] Li G., Yan Y.J., Huan Y., Zhou Y., Pang X.F. (2012). The influences of electromagnetic field with extremely low frequency on the characteristics of infrared spectrum of the sensitive tissue in rat. Spectrosc. Spectr. Anal..

[15] Li G., Li B., Yan Y.J., Yang S.F., Pang X.F. (2011). Calculation of equivalent electrical parameters in human tissue and its applications. Mater. Rev..

[16] Zhang Y.M., Zhou Y., Pang X.F. (2012). The Investigation of effects of pulse electromagnetic field on the fluorescence spectrum of serum in rat. Spectrosc. Spectr. Anal..

[17] Zhang Y.M., Li G., Yan Y.J., Pang X.F. (2011). Altered expression of matrix metalloproteinases and tight junction proteins with PEMF-induced BBB permeability change in rats. Biomed. Environ. Sci..

[18] Beebe S.J., Fox P.M., Rec L.J., Somers K., Stark R.H., Schoenbach K.H. (2002). Nanosecond pulsed electric field (nsPEF) effects on cells and tissues: Apoptosis induction and tumor growth inhibition. IEEE Trans. Plasma Sci..

[19] Lazzaro V.D., Capone F., Apollonio F., Borea P.A., Cadossi R., Fassina L., Grassi C., Liberti M., Paffi A., Parazzini M. (2013). A consensus panel review of central nervous system effects of the exposure to low-intensity extremely low-frequency magnetic fields. Brain Stimul..

[20] Apollonio F., Liberti M., Paffi A., Merla C., Marracino P., Denzi A., Marino C., d’Inzeo G. (2013). Feasibility for microwaves energy to affect biological systems via nonthermal mechanisms: A systematic approach. IEEE Trans. Microw. Theory Tech..

[21] Hofmann G.A., Dev S.B., Dimer S., Nanda G.S. (1999). Electroporation therapy: A new approach for treatment of head and neck cancer. IEEE Trans. Biomed. Eng..

[22] Kekez M.M., Savic P., Johnson B.F. (1996). Contribution to the biophysics of the lethal effects of electric field on microorganisms. Biochim. Biophys. Acta.

[23] Pang X.F., Zhang A.Y. (2001). Investigations of mechanism and properties of biological thermal effects of the microwave. China J. Atom. Mol. Phys..

[24] Davydov A.S. (1982). Biology and Quantum Mechanics.

[25] Davydov A.S. (1991). Solitons in Molecular Systems.

[26] Davydov A.S., Ermakov V.N. (1988). Soliton generation at the boundary of molecular chain. Phys. D.

[27] Davydov A.S. (1979). Solitons in molecular systems. Phys. Scr..

[28] Davydov A.S. (1991). The lifetime of molecular solitons. J. Biol. Phys..

[29] Davydov A.S. (1977). Solitons and energy transfer along protein molecules. J. Theor. Biol..

[30] Scott A.C. (1992). Davydov’s soliton. Phys. Rep..

[31] Scott A.C. (1982). Dynamics of Davydov’s soliton. Phys. Rev. A.

[32] Brown D.W., Ivic Z. (1989). Unification of polaron and soliton theories of exciton transport. Phys. Rev. E.

[33] Brown D.W., Lindenberg K., West B.J. (1987). Nonlinear density-matrix equation for the study of finite-temperature soliton dynamics. Phys. Rev. B.

[34] Guo B.L., Pang X.F. (1987). Solitons.

[35] Pang X.F. (1986). The transport of bio-energy in protein molecules. Chin. J. Biochem. Biophys..

[36] Christiansen P.L., Scott A.C. (1990). Davydov’s Soliton Revisited.

[37] Cruzeiro L., Halding J., Christiansen P.L., Skovgard O., Scott A.C. (1985). Temperature effects on Davydov soliton. Phys. Rev. A.

[38] Cruzeio-Hansson L. (1992). Mechanism of thermal destabilization of the Davydov soliton. Phys. Rev. A.

[39] Forner W. (1991). Quantum and disorder effects in Davydov soliton theory. Phys. Rev. A.

[40] Forner W. (1992). Quantum and temperature effects on Davydov soliton dynamics: Averaged Hamiltonian method. J. Phys. Condens. Matter.

[41] Forner W. (1991). Davydov soliton dynamics: Two-quantum states and diagonal disorder. J. Phys. Condens. Matter.

[42] Lomdahl P.S., Kerr W.C. (1985). Do Davydov solitons exist at 300 K?. Phys. Rev. Lett..

[43] Kerr W.C., Lomdahl P.S. (1987). Quantum-mechanical derivation of the equations of motion for Davydov solitons. Phys. Rev. B.

[44] Wang X., Brown D.W., Lindenberg K., West B. (1988). Alternative formulation of Davydov theory of energy transport in biomolecules systems. Phys. Rev. A.

[45] Wang X., Brown D.W., Lindenberg K. (1989). Quantum Monte Carlo simulation of Davydov model. Phys. Rev. Lett..

[46] Cottingham J.P., Schweitzer J.W. (1989). Calculation of the lifetime of a Davydov soliton at finite temperature. Phys. Rev. Lett..

[47] Schweitzer J.W. (1992). Lifetime of the Davydov soliton. Phys. Rev. A.

[48] Mettle B., Shaw P.B. (1988). Evolution of a molecular exciton on a Davydov lattice at T = 0. Phys. Rev. B.

[49] Ivic Z., Przulj Z., Kosti D. (2000). Decay and slowing down of the multiquanta Davydov-like solitons in molecular chains. Phys. Rev. E.

[50] Teki J., Ivic Z., Zekovi S., Przulj Z. (1999). Kinetic properties of multiquanta Davydov-like solitons inmolecular chains. Phys. Rev. E.

[51] Zekovi S., Ivic Z. (1999). Damping and modification of the multiquanta Davydov-like solitons in molecular chains. Bioelectrochem. Bioenerg..

[52] Pouthier V. (2003). Two-vibron bound states in alpha-helix proteins: The interplay between the intramolecular anharmonicity and the strong vibron-phonon coupling. Phys. Rev. E.

[53] Pouthier V., Falvo C. (2004). Relaxation channels of two-vibron bound states in α-helix proteins. Phys. Rev. E.

[54] Falvo C., Pouthier V. (2005). Vibron–polaron in α-helices. I. Single-vibron states. J. Chem. Phys..

[55] Falvo C., Pouthier V. (2005). Vibron–polaron in α-helices. II. Two-vibron states. J. Chem. Phys..

[56] Cruzeiro L. (2009). The Davydov/Scott model for energy storage and transport in proteins. J. Biol. Phys..

[57] Silva P.A.S., Cruzeiro L. (2006). Dynamics of a nonconserving Davydov monomer. Phys. Rev. E.

[58] Silva P.A.S., Cruzeiro-Hansson L. (2003). A reduced set of exact equations of motion for a non-number-conserving Hamiltonian. Phys. Lett. A.

[59] Pouthier V. (2008). Energy relaxation of the amide-I mode in hydrogen-bonded peptide units: A route to conformational change. J. Chem. Phys..

[60] Pouthier V., Tsybin Y.O. (2008). Amide-I relaxation-induced hydrogen bond distortion: Anintermediate in electron capture dissociation mass pectrometry of α-helical peptides?. J. Chem. Phys..

[61] Moritsugu K., Miyashita O., Kidera K. (2000). Vibrational energy transfer in a protein molecule. Phys. Rev. Lett..

[62] Fujisaki H., Zhang Y., Straub J.E. (2006). Time-dependent perturbation theory for vibrational energy relaxation and dephasing in peptides and proteins. J. Chem. Phys..

[63] Pang X.F. (1990). The properties of collective excitation in organic protein molecular system. J. Phys. Condens. Matter.

[64] Pang X.F. (1994). Comment the thermodynamic properties of α-helix protein: A soliton approach. Phys. Rev..

[65] Pang X.F. (1994). Nonlinear Quantum Mechanical Theory.

[66] Pang X.F. (1993). Properties of soliton in protein molecules with nonlinear nearest neighbour interaction. Chin. Sci. Bull..

[67] Pang X.F. (1993). The thermodynamic properties of the solitons excited in the protein molecules. Chin. Sci. Bull..

[68] Pang X.F. (1993). Quantum-mechamical method for the soliton transported bio-energy in protein. Chin. Phys. Lett..

[69] Pang X.F. (1993). Stability of the soliton excited in protein in the biological temperature range. Chin. Phys. Lett..

[70] Pang X.F. (1999). Influence of the soliton in anharmonic molecular crystals with temperature on Mossbauer effect. Eur. Phys. J. B.

[71] Pang X.F., Chen X.R. (2001). Distribution of vibrational energy- levels of protein molecular chains. Commun. Theor. Phys..

[72] Pang X.F. (2001). The effect of Raman scattering accompanied by the soliton excitation occurred in the molecular crystals. Phys. D.

[73] Pang X.F. (2000). An improvement of the Davydov theory of bio-energy etransport in the protein molecular systems. Phys. Rev. E.

[74] Pang X.F. (2001). The lifetime of the soliton in the improved Davydov model at the biological temperature 300 K for protein molecules. Eur. Phys. J. B.

[75] Pang X.F., Zhang H.W., Yu J.F., Feng Y.P. (2005). States and properties of the soliton transported bio-energy in nonuniform protein molecules at physiological temperature. Phys. Lett. A.

[76] Pang X.F., Luo Y.H. (2004). Stabilization of the soliton transported bio-energy in protein molecules in the improved model. Commun. Theor. Phys..

[77] Pang X.F., Feng Y.P. (2005). Quantum Mechanics in Nonlinear Systems.

[78] Pang X.F., Liu M.J. (2009). Features of Motion of Soliton Transported Bio-energy in aperiodic alpha-helix protein molecules with three channels. Commun. Theor. Phys..

[79] Pang X.F. (2009). The effects of damping and temperature of medium on the soliton excited in α-helix protein molecules with three channels. Mod. Phys. Lett. B.

[80] Pang X.F., Lui M.J. (2009). The influences of temperature and chain-chain interaction on features of solitons excited in α-Helix protein molecules with three channels. Int. J. Mod. Phys. B.

[81] Pang X.F. (2008). Influence of structure disorders and temperatures of systems on the bio-energy transport in protein molecules. Front. Phys. China.

[82] Pang X.F., Yu J.F., Lao Y.H. (2007). Combination effects of structure nonuniformity of proteins on the soliton transported bio-energy. Int. J. Mod. Phys. B.

[83] Pang X.F., Liu M.J. (2007). Properties of soliton-transported bio-energy in α-helix protein molecules with three channels. Commun. Theor. Phys..

[84] Pang X.F. (2007). Theory of bio-energy transport in protein molecules and its experimental evidences as well as applications (I). Front. Phys. China.

[85] Pang X.F., Zhang H.W. (2006). Theoretical investigation of properties of infrared absorption of α-helix protein molecules. Int. J. Infrared Millim. Waves.

[86] Pang X.F., Zhang H.W., Liu M.J. (2006). Influences of heat bath and structure disorder in protein molecules on the soliton transported bio-energy in the improved model. J. Phys. Condens. Matter.

[87] Pang X.F., Zhang H.W., Yu J.F., Luo Y.H. (2006). Influences of variations of characteristic parameters arising from the structure nonuniformity of the protein molecules on states of the soliton transported bio-energy in the improved model. Int. J. Mod. Phys. B.

[88] Pang X.F., Chen X.R. (2006). The properties of nonlinear energy-spectra of acetanilide. Int. J. Mod. Phys..

[89] Pang X.F., Yu J.F., Luo Y.H. (2005). Influences of quantum and disorder effects on solitons exited in protein molecules in improved model. Commun. Theor. Phys..

[90] Pang X.F., Zhang H.W. (2005). Changes of the Mössbauer effect caused by the excitation of the solitons in the organic molecular crystals at finite temperature. J. Phys. Chem. Solids.

[91] Pang X.F., Zhang H.W., Yu J.F., Luo Y.H. (2005). Thermal stability of the new soliton transported bio-energy under influence of fluctuations of characteristic Parameters at biological temperature in the protein molecules. Int. J. Mod. Phys. B.

[92] Pang X.F., Yu J.F., Liu M.J. (2010). Changes of properties of the soliton with temperature under influences of structure disorder in the α-helix protein molecules with three channels. Mol. Phys..

[93] Pang X.F., Chen X.R. (2002). Calculation of vibrational energy-spectra of α-helical protein molecules and its properties. Commun. Theor. Phys..

[94] Pang X.F., Chen X.R. (2002). Vibrational energy-spectra and infrared absorption of α-helical protein molecules. Chin. Phys. Lett..

[95] Pang X.F. (2009). Nonlinear Quantum Mechanics.

[96] Pang X.F., Xiao H.L., Cue G.P., Zhang H.W., Dong B. (2010). Experiment studies of properties of infrared absorption of biological tissues. Int. J. Infrared Millim. Waves.

[97] Pang X.F. (2012). The properties of bio-energy transport and influence of structure nonuniformity and temperature of systems on energy transport along polypeptide chains. Prog. Biophys. Mol. Biol..

[98] Pang X.F. (2012). The mechanism and properties of bio-photon emission and absorption in protein molecules in living systems. J. Appl. Phys..

[99] Pang X.F. (2011). The theory of bio-energy transport in the protein molecules and its properties. Phys. Life Rev..

[100] Pang X.F. (2011). Correctness and completeness of the theory of bio-energy transport. Phys. Life Rev..

[101] Pang X.F. (2014). The checkout and verification of theory of bio-energy transport in the protein molecules. Biophys. Rev. Lett..

[102] Pang X.F. (2014). The features of infrared spectrum of bio-polymer and its theoretical investigation. Int. J. Mod. Phys. B.

[103] Su X.D., Jin F.J. (2011). Davydov-Pang model: An improved Davydov protein soliton theory. Phys. Life Rev..

[104] Liang S.D. (2011). Physical insights to the bio-energy transport in the protein molecules. Phys. Life Rev..

[105] Tao S. (2011). The function of soliton on bio-energy transport in the protein molecules. Phys. Life Rev..

[106] He N.Y. (2011). An important biological theory—Solving the transport of bio-energy in living systems. Phys. Life Rev..

[107] Stiefel J. (1965). Einfuhrung in die Numerische Mathematic.

[108] Atkinson K.E. (1987). An Introdution to Numerical Analysis.

[109] Kumar A., Singh G. (2007). Measurement of dielectric constant and loss factor of the dielectric material at microwave frequencies. Prog. Electromagn. Res..

[110] Clifford E.F., Prilusky J., Silman I., Sussman J.L. (2007). A server and database for dipole moments of proteins. Nucleic Acids Res. Adv..

[111] Antosiewicz J. (1995). Computation of the dipole moments of proteins. Biophys. J..

[112] Marracino P., Amadei A., Apollonio F., d’Inzeo G., Liberti M., diCrescenzo A., Fontana A., Zappacosta R., Aschi M. (2011). Modeling of chemical reactions in micelle: Water-mediated, Keto-Enol Interconversion as a case study. J. Phys. Chem..

[113] Careri G., Buontempo U., Galluzzi F., Scott A.C., Gratton E., Shyamsunder E. (1984). Spectroscopic evidence for Davydov-like solitons in acetanilide. Phys. Rev. B.

[114] Careri G.A., Gransanti A., Rupley J.A. (1988). Critical exponents of protonic percolation in hydrated lysozyme powders. Phys. Rev. A.

[115] Careri G.A., Gratton E.A., Shyamsunder E. (1988). Fine structure of the amide-I bond in acetanilide. Phys. Rev. A.

[116] Careri G.A., Buonttempo U., Caeta F., Scott A.C. (1983). Infrared absorption in acetanilide by solitons. Phys. Rev. Lett..

[117] Careri G.A., Eilbeck J.C. (1985). Stability of solutions of the discrete self-trapping equation. Phys. Lett. A.

[118] Scott A.C. (1990). Davydov’s soliton revisited. Phys. D.

[119] Scott A.C., Gratton E., Shyamsunder E., Careri G. (1985). IR overtone spectrum of the vibrational soliton crystalline acetanilide. Phys. Rev. B.

[120] Scott A.C., Bigio I.J., Johnston C.T. (1989). Polarons in acetanide. Phys. Rev. B.

[121] Alexander D.M., Krumbansl J.A. (1986). Localized excitation in hydeogen-bonded molecular crystals. Phys. Rev. B.

[122] Alexander D.M. (1985). Analog of small Holstein polaron in hydrogen-bonded amide systems. Phys. Rev. Lett..

[123] Weaver R.F. (2002). Molecular Biology.

[124] Chen H.L. (1998). Structure and Function of Biomacromolecules.

[125] Pang X.F. (2003). Molecular Bio-Physics.

[126] Susi H., Ard T.S., Carroll J. (1971). The infrared spectrum and water binding of collagen as a function of relative humidity. Biopolymers.

